# Supplementation with endogenous healthy gut metabolites reverses the disruptions of in vitro and ex vivo epithelial functions induced by fecal content from IBD patients

**DOI:** 10.1080/19490976.2025.2597568

**Published:** 2025-12-15

**Authors:** Haya Abbas-Egbariya, Lubna Elwahidi, David Jessula Levy, Tzipi Braun, Nina Levhar, Rotem Hadar, Gilat Efroni, Maya Granot, Yael Leichtmann-Bardogoo, Ben M. Maoz, Batia Weiss, Ohad Gal-Mor, Bella Agranovich, Ifat Abramovich, Lee Denson, Shomron Ben-Horin, Kelli L. VanDussen, Amnon Amir, Yael Haberman

**Affiliations:** a Sheba Medical Center, Tel-Hashomer, affiliated with the School of Medicine, Faculty of Medical and Health Sciences, Tel-Aviv University, Tel Aviv, Israel; b Gray Faculty of Medicine, Faculty of Medical and Health Sciences, Tel-Aviv University, Tel-Aviv, Israel; c Department of Biomedical Engineering, and Sagol School of Neuroscience at Tel Aviv University, Tel Aviv, Israel; d Sagol School of Neuroscience, Tel-Aviv University, Tel-Aviv, Israel; e The Center for Nanoscience and Nanotechnology, Tel Aviv University, Tel Aviv, Israel; f The George S. Wise Faculty of Life Sciences, Tel Aviv University, Tel Aviv, Israel; g The Infectious Diseases Research Laboratory, Sheba Medical Center, Tel-Hashomer, Israel; h Department of Clinical Microbiology and Immunology, Tel Aviv University, Tel Aviv, Israel; i Laura and Isaac Perlmutter Metabolomics Center, Technion-Israel Institute of Technology, Bat Galim, Haifa, Israel; j The Ruth and Bruce Rappaport Faculty of Medicine, Technion-Israel Institute of Technology, Bat Galim, Haifa, Israel; k Cincinnati Children’s Hospital Medical Center, Department of Pediatrics, University of Cincinnati College of Medicine, Cincinnati, OH, USA

**Keywords:** Inflammatory bowel disease (IBD), metabolites, epithelia, microbiome

## Abstract

Despite epithelial involvement in inflammatory bowel disease (IBD) pathogenesis and the gaps in treatment goals with existing immune-directed therapy, epithelial-directed interventions are unavailable. Using patient-based models, we aimed to identify bioactive endogenous metabolites that can improve IBD epithelial dysfunction, are generally regarded as safe, and can enhance epithelial homeostasis. We pooled fecal material from subjects with and without IBD to capture patient heterogeneity and analyzed the fecal contents for microbiome composition and metabolomics. Epithelial cells (Caco-2 cells and patient-derived colonoids) were cultured, and fecal material was applied apically to replicate the gut's physiological orientation. Measurable epithelial outputs included epithelial proinflammatory signals, integrity, and cellular ATP levels. We show that fecal content pools from several independent IBD patients disturb epithelial functions significantly more than does the fecal content from controls. Improved epithelial readouts in the functional patient-based models were linked with several gut metabolite levels, and these findings were further validated in an independent published human biospecimen multi-omics in vivo cohort. This guided the supplementation of five prioritized metabolites (azelate, pyridoxal, fructose-6-phosphate, galactose 1-phosphate, and ribose 5-phosphate) into the IBD fecal content, which reversed the related IBD epithelial dysfunction. We streamline a proof-of-concept pipeline for the prioritization of epithelial-targeted metabolite interventions that can direct safe future novel adjunct interventions.

## Introduction

Gut epithelial functions are severely affected by inflammatory bowel disease (IBD), along with the activation of the immune system. However, current treatments focus primarily on suppressing the immune response rather than enhancing gut epithelial functions and achieve sustained remission in fewer than 50% of patients. The pathological interactions between metabolites, gut bacteria, and gut epithelial cells in IBD have been emphasized and refined using advancing omics tools.^1-3^ The PROTECT UC cohort identified marked alterations in epithelial functions, including the suppression of epithelial mitochondrial genes and function^4^ and altered epithelial lipid metabolism.^4^ The multi-omics SOURCE CD cohort captured alterations in epithelial lipid and mitochondrial function and the induction of epithelial immune functions linked with microbial and metabolite signals.^3^ Epithelial barrier dysfunction and increased intestinal permeability persist even during disease remission.^5^^,^^6^ A paradigm shift that combines therapies targeting epithelial tissue with immune suppression may enhance mucosal healing and improve patient outcomes, an unmet clinical need in IBD. Metabolites derived from the host or microbiota include endogenous bioactive compounds that can support epithelial functions. These compounds are generally considered safe and can be easily applied. A previous attempt to use metabolites such as short-chain fatty acid topical treatments for distal ulcerative colitis demonstrated feasibility and effectiveness,^7^ highlighting the potential of such an approach.

Non-human models do not fully address the complexity and variability of human factors influenced by diet and the gut microbiota, highlighting the need for preclinical patient-based models. Here, we employed a functional culturing system focusing on host cell–metabolite–microbiome interactions that compares the impact of fecal content from IBD vs. control subjects on epithelial readouts and uses the system to prioritize functionally bioactive metabolites that improve epithelial functions. To overcome challenges associated with coculturing aerobic epithelia and fecal content with predominantly anaerobic microbiota,^8^^,^^9^ we processed fecal samples by separating the supernatant (containing metabolites), heat-killing the bacteria, and rejoining the samples for culture with epithelia. To capture diverse patient heterogeneity, we prioritized and pooled fecal materials from groups of ulcerative colitis (UC), Crohn’s disease (CD), and healthy subjects. We then tested the effects on two epithelial model systems: Caco-2 cells and differentiated mucosal biopsy-derived colonoids from several human control subjects. Using this approach, we demonstrated more pronounced attenuation of epithelial functions with fecal content obtained from IBD patients than controls without IBD. This process guided the prioritization of metabolites that were then supplemented into IBD fecal content and subsequently enhanced epithelial health.

## Materials and methods (see Supplementary methods)

**Patient sampling**. Fecal samples, demographics, clinical and laboratory results, and disease characteristics were collected from CD and UC patients and healthy volunteers (10 subjects per group in pools #1, and 5 per group in pools #2 and #3). Oral samples were collected from 10 healthy subjects. Biopsy samples for organoid cultures (*n* = 5) were obtained during endoscopy from non-IBD controls as part of routine medical care.^10^^,^^11^ The Sheba Institutional Review Board approved the protocol and safety monitoring plan (IRB #1514). Informed consent was obtained from each participant.

**Figure 1.**
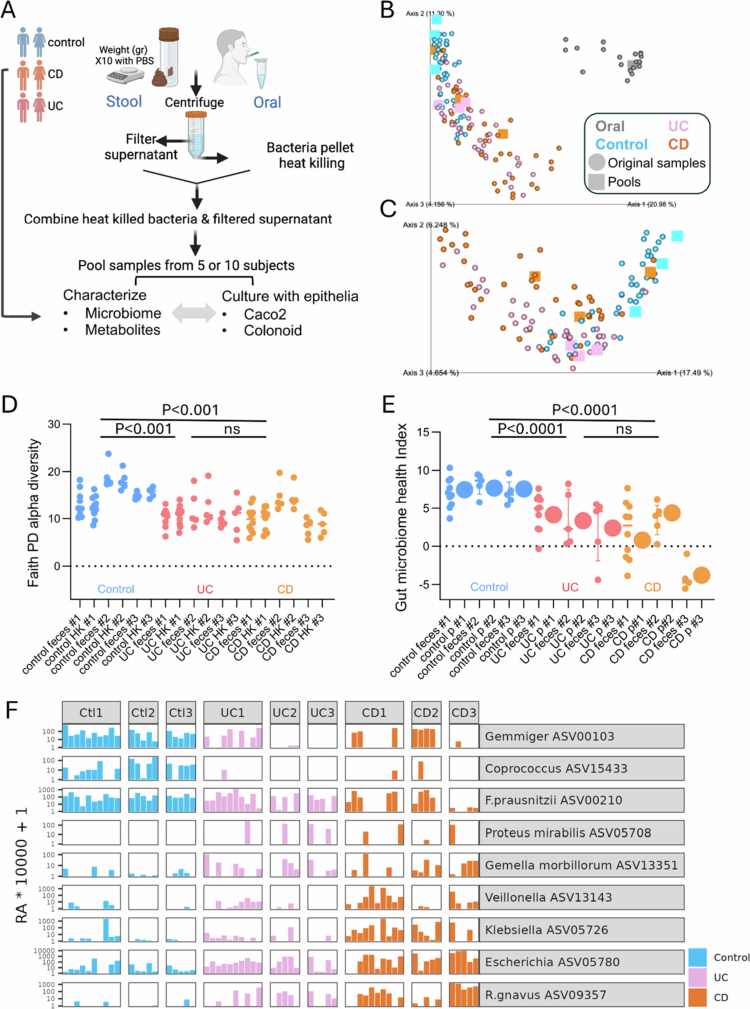
Development and characterization of heat-inactivated fecal pooled samples. (A) Schematic overview of generating heat-inactivated bacterial pools from fecal samples collected from healthy subjects (controls), patients with Crohn's disease (CD) and ulcerative colitis (UC) patients, and oral (saliva) samples collected from healthy subjects. (B, C) Unweighted UniFrac PCoA graph for all the 16S bacterial samples (fecal and oral, B) and only fecal samples (C), including the pools that were processed in the pipeline in A., which are marked as squares. (D) Faith alpha diversity values for 16S samples before processing (feces) and after processing (heat-killed, HK) for pools #1, #2, and #3. (E) Gut microbiome health indices of individual fecal samples and heat-inactivated pools (#1, #2, and #3). *P*-values are from t-tests between unprocessed fecal samples per group, including Crohn’s disease (CD) vs. controls, ulcerative colitis (UC) vs. controls, and UD vs. CD for D-E (*n* = 20 samples per group). (F) Normalized relative abundance (RA × 10,000 + 1) of specific ASVs and their taxonomies, which resulted from Maaslin2 differential abundance analyses between IBD and control fecal samples, controlling for age and gender (FDR < 0.25, top 20 ASV in each direction in S1B, and the full list in Dataset S1, where each ASV number is related to a specific sequence). The results are grouped into three diagnostic categories: controls (Ctl p#1−3), ulcerative colitis (UC p#1−3), and Crohn’s disease (CD p#1−3).

**Fecal sample preparation (see the scheme in Figure 1).** One gram of each fecal sample was suspended in 10 ml of PBS. The supernatants were filtered to remove debris. Samples were centrifuged, and the fecal supernatants (Sup.) containing non-cellular molecules and metabolites were separated from the bacterial pellets. For heat inactivation (heat-killed bacteria, HK), the pellets were then incubated in 1 ml of PBS at 85 °C for 60  min for heat inactivation (heat-killed bacteria, HK). The HK fraction was then rejoined with the original fecal Sup (keeping the same 1:10 ratio between the HK fraction and the fecal Sup). Oral (saliva) samples were processed similarly, starting with ~1 ml of saliva/oral fluid, without adding PBS. The fecal content (joined HK and Sup, 1:10 in PBS) was mixed 1:2 in the media when incubated with Caco-2 cells, and 1:4 in the media when incubated with colonoids. The oral pool was diluted 1:2 in all experimental assays. Each pool consisted exclusively of either fecal or oral samples. The purpose of using pools is to capture heterogeneity while also reducing the number of comparisons.

**16S rRNA sequencing and analyses**. Microbial characterization was conducted using the variable region 4 (V4, 515F/806R primers) PCR of 16S rRNA^12-14^ and the Illumina MiSeq. Reads were processed in QIIME 2 2023.7.^15^^,^^16^ Amplicon sequence variants (ASV) detection was performed using Deblur.^17^ ASV taxonomic classification was assigned using the 2022.10 Greengenes2 database.^18^ For ASVs that did not have an exact match in the database, a naive Bayes-fitted classifier trained on the same 2022.10 Greengenes2 database was used instead. Taxonomy assigned by 16S is indicated by the specific ASV number, and the sequence associated with each number is in Dataset S1. All the samples were rarefied to 3000 reads for Faith’s phylogenetic diversity^19^ alpha and unweighted UniFrac beta diversity. The resulting distance matrix was used to perform a principal coordinate analysis (PCoA). Health index: Per-sample health index was calculated as previously described:^20^ for each sample, the log of the ratio of health-associated ASVs bacteria (97) to disease-associated ASVs bacteria (31) was calculated and defined as the health index (with higher values indicating a better health-associated microbiome). Multivariate association with linear models (MaAsLin2 R package 1.8.0^21^) was used to identify differentially abundant ASVs between IBD and controls, controlling for age and gender.

**Metabolomics.** Liquid chromatography‒mass spectrometry (LC‒MS) analysis was conducted as described^22^ using Dionex Ultimate ultra-high-performance liquid chromatography (UPLC) system coupled to an Orbitrap Q-Exactive Mass Spectrometer (Thermo Fisher Scientific). Peak areas of metabolites were determined using MZmine 2.53^23^ based on the exact mass of the singly charged ions (m/z), and the retention time of the metabolites was predetermined on the pHILIC column by analyzing an in-house mass spectrometry metabolite library that was built by running commercially available standards (*n* = 549). Control, UC, and CD pools (p#1−3) and the fecal Sup. were run in a single batch. Spearman's rank correlation (with a FDR multiple hypothesis correction threshold of 0.25) was applied using Calour to examine the relationships between individual metabolite abundances and two key metrics: organoid ATP levels and transepithelial electrical resistance (TEER) in the Caco-2 system. Before analysis, metabolite abundance data underwent filtering of spike-in metabolites, and total sum scale (TSS) normalization, and filtering to keep only metabolites significantly different between the IBD and control samples (permutation-based non-parametric mean-rank test with dsFDR ≤ 0.1, as implemented in Calour). Metaboanalyst 5.0^24^^,^^25^ was used for pathway enrichment analyses (Suppl. Dataset S2). The concentrations used for G-MB low were: azelate 1 µM, pyridoxal 0.1 µM, fructose-6-phosphate 1 µM, galactose 1-phosphate 0.1 µM, and ribose 5-phosphate 0.002 µM, and for G-MB high: azelate 10 µM, pyridoxal 10 µM, fructose−6-phosphate 10 µM, galactose 1-phosphate 1 µM, and ribose 5-phosphate 1 µM.

**Epithelial models**. Crypt-derived organoid preparation was performed as described^26^^,^^27^ from two biopsies collected from the recto-sigmoid region during a routine colonoscopy. For the indicated experiments, the colonoids were seeded as monolayers and allowed to differentiate for 72 h before various interventions were applied and in a 3D apical-out configuration^28^ for florescence staining in 3D. Caco-2 human colon carcinoma cell lines from the American Type Culture Collection (Manassas, VA, USA) were maintained under standard culture conditions. IFNγ (40 ng/ml), TNFα (20 ng/ml), and LPS (100 ng/ml) were applied. Fecal extract pools were applied apically. The transepithelial electrical resistance (TEER) was determined using Millicell ERS-2 voltohmmeter (Millipore). Each insert was measured three times, and average values were corrected for background TEER and the surface area of the insert to obtain the net-area resistance in Ω∗cm2. The CellTiter-Glo® Luminescent Cell Viability ATP levels Assay (Promega, G7571) was used according to the manufacturer’s protocol. For live imaging, colonoids were incubated with Hoechst (Thermo Fisher 33342), and imaging was performed using a fluorescence microscope (Olympus ix83) at 4x magnification and a confocal microscope at 20x magnification (Olympus ix83). Staining of fixed cells was performed using anti-ZO1 antibodies (Thermo Fisher 33-9100), DAPI Fluoromount G (SouthernBiotech 0100), and actin Phalloidin-iFluor 488 (Abcam ab176753). CXCL1 (R&D Systems, catalog number DY453) levels were measured using specific sandwich ELISA kits according to the manufacturer’s protocol. For cellular *CXCL1* mRNA expression, RT-qPCR was performed using CXCL1-specific primers (forward: 5′-GCAGCAGTCAGTGAGTCTCTTC-3′ and reverse: 5′-GGGGACTTCACGTTCACACT-3′). For mRNA expression *of CXCL8* (IL-8), RT-qPCR-specific primers (forward: 5′-GGAGAAGTTTTTGAAGAGGGCTGAGAAT-3’ and reverse: 5′-CAGACCCACACAATACATGAAGTGTTG-3′), DUOX2 RT-qPCR-specific primers (forward: 5′- ACGCAGCTCTGTGTCAAAGGT-3’ and reverse: 5′- TGATGAACGAGACTCGACAGC-3′), and TGM2 RT-qPCR-specific primers (forward: 5′- GGCATGGTCAACTGCAAC-3′ and reverse: 5′- CAGCACTGGCCATACTTGAC-3′).

**Metabolite cross-validation**. Data from our previously published multi-omics study,^3^ which characterized terminal ileum transcriptomics and metabolomics profiles between CD patients and controls, were used to validate the association between 18 metabolites and epithelial health. These metabolites were identified as significantly correlated with epithelial ATP levels or TEER in our culture model. To identify coordinated gene expression patterns, modules of co-expressed genes were constructed using the WGCNA package (version 1.72-1) in R.^29^ The correlation between metabolite levels and disease-associated gene modules was then assessed, following established methods.^3^ Benjamini‒Hochberg FDR correction was utilized only to the metabolites and modules tested here.

**Statistical analysis**. One-way ANOVA with Šídák's multiple comparisons testing and t-tests were used for normally distributed variables, and the Mann‒Whitney U test for variables that did not meet the criteria for normality. Categorical variables were reported as frequencies and percentages, with associations analyzed using the chi-square test. *p < 0.05, **p < 0.01, ***p < 0.001, **** *p* < 0.0001. Analyses were performed with GraphPad Prism v10.1.2.

**Data availability**. The processed 16S data are available as Dataset S1. The 16S amplicon sequencing dataset was deposited as BioProject PRJNA1216163. Metabolite prioritizations are available as Dataset S2.

## Results

### Establishing a model system based on whole human IBD and control fecal samples

The culturing model is based on human fecal samples cultured with human-derived epithelia to assess causal effects on defined epithelial readouts. We processed fecal samples from patients with CD and UC and controls without IBD. Our pipeline included separating the fecal supernatant (Sup. which contains metabolites), heat-killing (HK) the bacteria, and rejoining the samples (Figure 1A, see methods). Samples obtained from healthy volunteers' oral cavities were similarly processed as non-fecal bacterial and metabolite sources.

To capture heterogeneity across patients, we initially prioritized and pooled together fecal material from CD, UC, and healthy control subjects (Table S1, pool #1, *n* = 10 subjects per group). Prioritization of pool #1 samples was based on our published microbial health index,^20^matching gender and age between pools. This microbial index is based on analyses of 12,838 subjects from 59 disease cohorts that demonstrated a predominant shared signature across diseases, including IBD.^20^ This index uses the log of the ratio of health- to disease-associated amplicon sequence variants (ASVs) bacteria with higher values indicating a better health-associated microbiome. The median age of the participants who submitted fecal samples was 23 y (IQR 17−28), 57% were male, and the median BMI was 21.6 (IQR 19−25). Within the UC and CD patients included in pool #1, 50% and 70% had clinically active disease, respectively, with median fecal calprotectin levels of 420 in the UC group and 1000 in the CD group. To ensure reproducibility and broader signal generalization, we expanded the fecal pool repertoire to include two additional independent pools per group (*p* #2 & #3 from CD, UC, and healthy controls), each containing samples from 5 independent subjects (Table S2). The median age of participants who submitted fecal samples for *p* #2 and #3 was 36 y (IQR 25−46); 60% were male, and the median BMI was 23 (IQR 20−25). Within *p* #2 and #3, 80% of UC and 20% of CD had clinically active disease with the indicated fecal calprotectin levels.

16S sequencing analyses revealed that the overall bacterial composition (Figure 1B,C), Faith alpha diversity (Figure 1D), and the respective health index (Figure 1E) of the original fecal/oral material, the processed HK samples, and the rejoined and pooled fractions were preserved after processing, as in the original samples. The IBD samples showed reduced alpha diversity and health index versus control samples. We verified that similar quantities of bacterial DNA were present in pooled oral and fecal samples through 16S qPCR analysis (Figure S1A). Using multivariate analysis (Maaslin2) and controlling for age and gender, we identified 23 ASVs that were more abundant in IBD, and 97 in the control samples (FDR < 0.25, Figure 1F, S1B, Dataset S1, where each ASV number was related to a specific sequence). As previously shown in other cohorts,^11^^,^^30-32^ the abundances of the ASVs linked with *Escherichia coli* (ASV05780), *Gemella morbillorum* (ASV13351), *Veillonella parvula/dispar* (ASV13143), *Ruminococcu gnavus* (ASV09357), and *Klebsiella* (ASV05726) were more prevalent in the IBD samples, while *Proteus mirabilis* (ASV05708) was present in the IBD samples but not in the control samples (Figure 1F, S1B, Dataset S1). On the other hand, the abundances of the ASVs *Coprococcus eutactus* (ASV15433), *Faecalibacterium prausnitzi* (ASV00210), and *Gemmiger formicilis* (ASV00103) were higher in the control samples. A specific pathogen search revealed that only four subjects (7%) had ASVs linked with *Clostridium difficile* [1/20 controls (P#3), 1/20 UC patients (P#2), 2/20 CD patients (p#1 and p#3), Figure S1C], and no ASVs linked with *Campylobacter jejuni* or *Salmonella* were detected (Dataset S1). In addition, we demonstrated that the processed samples, before incubation with the epithelial cells, contained only negligible levels of CXCL1 and minimal amounts of ATP (Figure S1D, E), as these levels were measured during the culturing with epithelial cells as readouts.

#### IBD fecal samples increased inflammatory response and decreased intestinal integrity in Caco-2 cells compared to control samples

**Figure 2.**
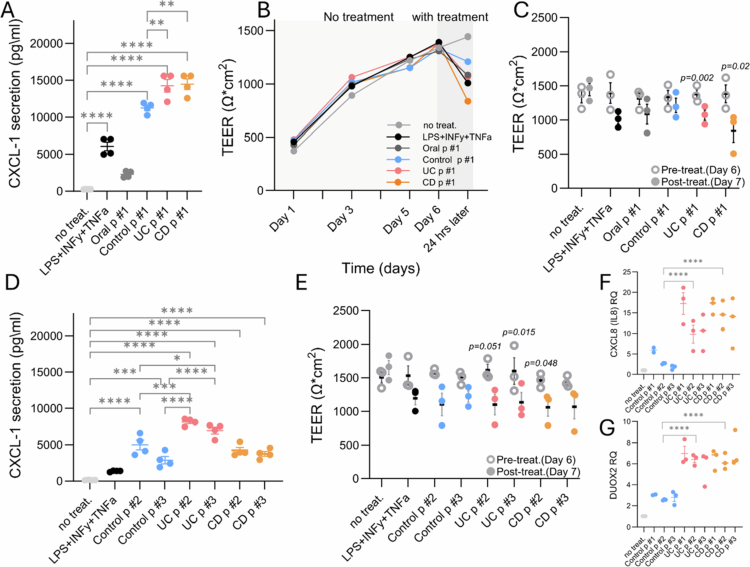
Culturing fecal content and Caco-2 cells shows disease-specific effects. (A) CXCL1 levels were measured in the culture media 24 h after exposing Caco-2 epithelial cells to fecal pools or inflammatory triggers (LPS, IFNγ, and TNFα), heat-inactivated fecal content from the control, UC, and CD pools #1. The values were normalized to those of the untreated control group; the lines indicate the median and inter-quartile range (IQR). One-way ANOVA with Šídák's multiple comparisons test was used for comparison. (B,C) Transepithelial electrical resistance (TEER) measurements of Caco-2 cells seeded on transwells (5 × 10⁵ cells/well). TEER values were recorded daily from the start of culture (day 1) through treatment administration on days 6 and 7 (24 h post-treatment, B). Summarized results on day 6 (pre-intervention) and day 7 (24 h post-interventions) are shown as the median (IQR) for p#1, with paired t-tests comparing values before and 24 h after treatment (C). TEER values were calculated using the equation: TEER (Ω × cm²) = net resistance (Ω) × membrane area (cm²). (D) CXCL1 levels as in A but with fecal pools #2 and #3. (E) TEER values as in C but with incubation with p#2 and #3 (one-way ANOVA with Šídák's multiple comparisons test was used for comparison). (F, G) CXCL8 (IL8) (F) and DUOX2 (G) mRNA levels (RQ normalized to GAPDH) are shown 24 h after exposing Caco-2 epithelial cells to the non-IBD and IBD fecal content pools. * *p* < 0.05, **p < 0.01, ***p < 0.001, ****p < 0.0001.

The processed fecal contents were cultured with Caco-2 monolayer cells. The measurable outputs included cellular ATP levels, epithelial integrity measured by trans-epithelial electrical resistance (TEER), epithelial secretion of CXCL1 into the medium, and *CXCL1* mRNA expression. CXCL1 was chosen as a representative proinflammatory chemokine because it was shown to be increased in IBD and to mediate immune and neutrophil activation.^33-35^ Culturing Caco-2 cells with the fecal material from p#1 controls, UC and CD, showed a significant increase in CXCL1 secretion into the media (Figure 2A) and *CXCL1* mRNA expression (Figure S1G, H) in comparison to cells without treatment, cells treated with an inflammatory triggers mix (IFN-*γ*, TNFα, and LPS), and cells cultured with pooled oral samples (One-way ANOVA with Šídák's multiple comparisons test, *p* < 0.05). The UC p#1 and CD p#1 increased CXCL1 proinflammatory chemokine secretion more than control p#1 (Figure 2A showing biological replicates), and higher *CXCL1* mRNA expression was noted upon incubation with UC vs. control p#1 (Figure S1G). The UC and CD pools significantly decreased epithelial integrity, as measured by TEER (paired t-test between pre-treatment days 6 and post-treatment day 7, *p* < 0.05, Figure 2B, C), without affecting Caco-2 cellular ATP levels (Figure S1F), which are also linked with epithelial metabolic function.^36^^,^^37^ Validation was conducted using fecal pools (p#2 and p#3) from different IBD and control subjects. While there are variations between the different human pools, the overall signals showed similar trends (Figure 2D, E); we noted a significant increase in CXCL1 secretion into the media (*p* < 0.05), which was highest with pools obtained from UC patients (Figure 2D) and higher *CXCL1* mRNA expression with CD and UC p#2−3 compared to control (Figure S1H). Higher *CXCL8* (IL8) and *DUOX2* mRNA expression was further noted in the IBD fecal content vs. non-IBD controls (Figure 2G-H). Interestingly, higher TGM2, which we and others linked to IBD^10^^,^^38^ (including CD, UC) and metabolic dysfunctions in IBD^38^, was noted in the IBD patients than in the non-IBD controls (Figure S1I). UC and CD pools p#2−3 also decreased epithelial integrity, as measured by TEER, which significantly decreased with UC p#3 and CD p#2 (Figure 2E). Staining with ZO1 (TJP1, Figure S2A) showed variable but more pronounced tortuous, often discontinuous morphology and staining for actin (Figure S2B) showed visibly fragmented areas^39^ when the cells were treated with IBD vs. control fecal content.

### IBD fecal content disturbs primary colonoid ATP levels more than control fecal content

**Figure 3.**
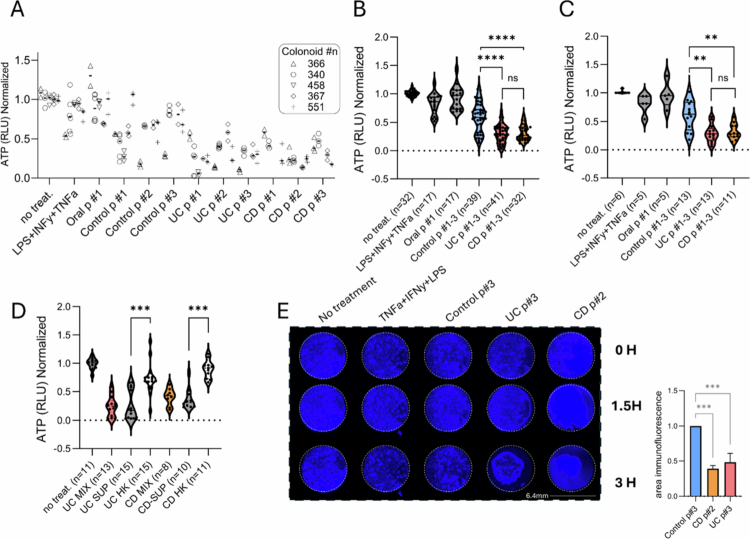
Culturing fecal content from IBD patients showed more harmful effects on epithelial ATP levels than fecal content from control subjects. (A–C) Five colonoids (derived from 5 non-IBD subjects) were seeded as monolayers, differentiated for 72 h, and exposed to various interventions for 5 h. ATP levels were assessed using the ATP-Glo cell luminescence kit, with ATP levels normalized to untreated controls. (A) Normalized ATP levels are shown by pool type (oral p#1 and control/UC/CD p#1−3), and the colonoid lines used are indicated by shape. (B, C) Normalized ATP levels (from Figure 4) aggregated by pooled diagnosis for all measurements (B) and as average normalized ATP levels (C), grouped by pooled diagnosis. (D) Normalized ATP levels after the incubation of organoids with the combined fecal supernatant and heat-killed bacteria (MIX), fecal supernatant only (SUP), and heat-killed bacteria only (HK). (E) Colonoids stained with Hoechst were imaged using a fluorescence microscope at 4×  magnification. Images were captured at baseline (0 h) and after 1.5 and 3 h of exposure to the indicated fecal supernatant-only interventions. Mann‒Whitney tests between pairs of UC, CD, and controls were used for comparison in B-C, and between HK and SUP in D. The bar graph shows the quantification of signals in the IBD pools normalized to the control p#3 (*n* = 3 experiments, t-test). * *p* < 0.05, **p < 0.01, ***p < 0.001, ****p < 0.0001, ns *p* > = 0.05.

We then tested the effects of the fecal content on a more physiological primary epithelial culture. We generated rectal biopsies-derived epithelial organoid cultures (colonoids) from five non-IBD controls (Table S3). The colonoids were seeded as monolayers and allowed to differentiate for 72 h before various interventions were applied for an additional 5 h, after which we quantified ATP levels. Incubation with inflammatory triggers (IFNγ, TNFα, and LPS) and oral sample #1 had minimal effects on epithelial ATP levels (Figure 3A−C). Notably, processed fecal content from patients with UC or CD had a much greater effect on reducing colonoid ATP levels compared to fecal content obtained from healthy controls. This trend was consistent whether we analyzed all data points from pools #1−3 (Figure 3B) or averaged the measurements for each colonoid (the Mann‒Whitney test between pairs of UC, CD, and controls were used for comparison, Figure 3C). To determine whether the observed effects on ATP levels were due to heat-killed (HK) bacteria or the fecal supernatant, we cultured the colonoids with each fraction separately and compared these effects to those seen with the combined material. Notably, the impact on the UC and CD fecal samples was more significant when the fecal supernatant was used than when the HK bacteria were used (Mann‒Whitney test, Figure 3D). Live fluorescence microscopy confirmed phenotypic changes when culturing the epithelial cells with fecal content from IBD patients compared to treatment with fecal content from healthy controls. This was evidenced by the detachment of monolayer cells from the surface and cell clumping with the IBD samples, indicative of effects on cell viability (Figure 3E and Figure S3). Additional staining of flipped apical-out organoids incubated with fecal content from non-IBD and IBD applied in the apical part also revealed alterations in ZO1 and actin phalloidin stains across several colonoids exposed to the IBD fecal pools (Figure S4).

### Metabolite supplementation, guided by functional prioritizations, into IBD fecal content enhances epithelial functions

**Figure 4.**
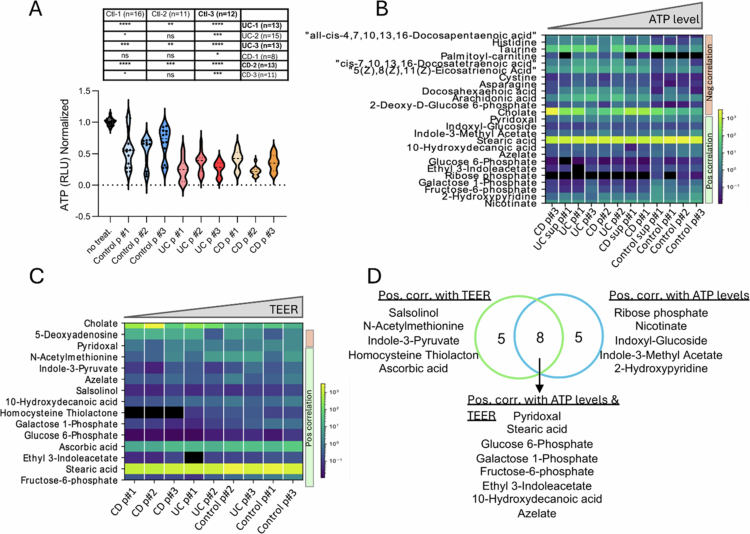
Prioritized metabolites linked with improved epithelial functions. (A) Normalized colonoids ATP levels aggregated by pools p#1−3 and by control, UC, and CD states, showing variations in effects within the disease pool on epithelial ATP levels. One-way ANOVA with Šídák's multiple comparisons tests was used for UC vs. control pools #1−3 and CD vs. control pools p#1−3; *p-* values are summarized in the table. (B, C) Heatmaps summarizing Spearman correlations between disease-associated metabolite levels and colonoids ATP levels (B) and TEER (C) (FDR < 0.25). The colors represent normalized metabolite levels samples are sorted by ATP levels or TEER on the X-axis. (D) Venn diagram highlighting metabolites correlating with TEER and ATP levels.

By utilizing the different compositions of each pool, we tested for effect variation between the different pools. Fecal samples from healthy individuals and those with UC and CD showed differences in their effects on the experimental outcomes (Figures 2 and 4A). Among the healthy control samples, control pool #3 had the lowest impact on colonoid ATP levels and the weakest effect on CXCL1 secretion and integrity (TEER) in Caco-2 cells. In contrast, UC pools #1 and #3, along with CD pool #2, demonstrated the most pronounced impacts on ATP levels and Caco-2 epithelial integrity, as indicated by TEER.

**Figure 5.**
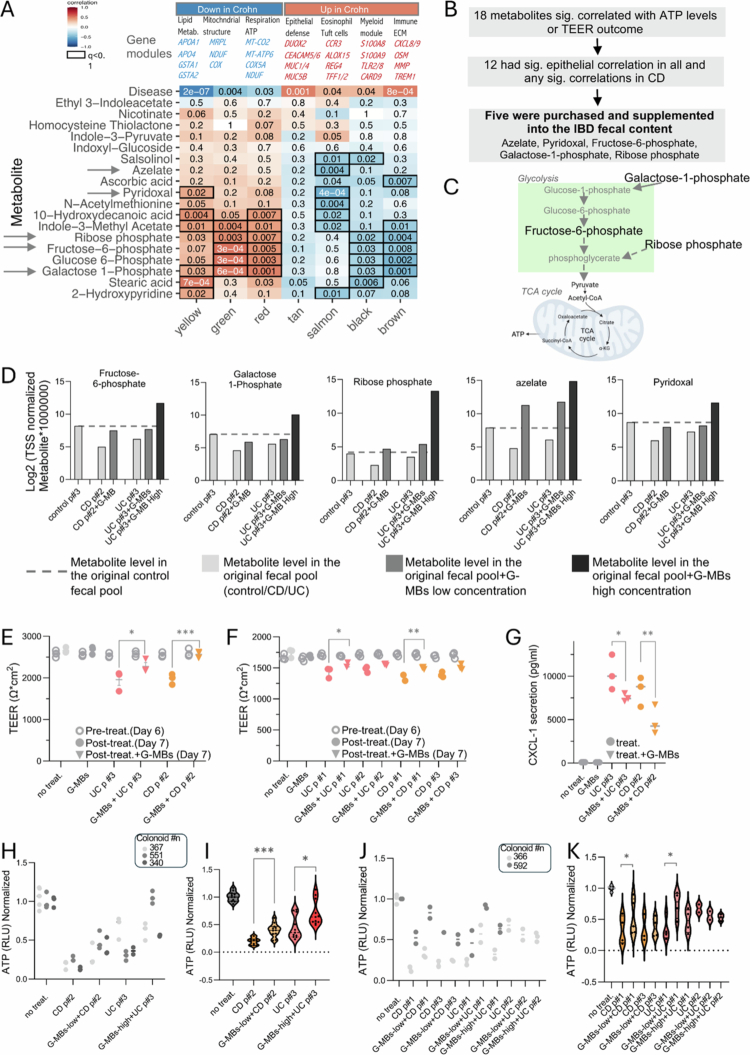
Supplementing IBD fecal content with prioritized metabolites improved epithelial functions. (A) In vivo cross-validation between metabolite levels and mucosal transcriptomics epithelial modules (see Methods) based on a previously published multi-omics dataset,^3^ here specifically focusing on 18 metabolites that were significantly linked (FDR ≤ 0.1) to epithelial ATP levels (B) or TEER (C) in culture. The heatmap represents the correlation between each mucosal transcriptomics gene module and metabolite levels. Disease-associated modules were used (*p* < 0.05). The numbers represent the correlation *p*-value and the color represents the coefficient for each comparison. Correlations with Benjamini‒Hochberg FDR values ≤ 0.1 are marked with black squares. The arrows indicate the 5 metabolites selected for the supplementation. B. Schematic representation of the prioritization process for selecting potential beneficial “good metabolites” (G-MBs) that will be supplemented into the IBD fecal content. C. Schema showing connections between fructose-6-phosphate, galactose-1-phosphate, and ribose phosphate and ATP production. (D) Bar graph showing normalized metabolite levels (total sum scaling (TSS) normalization) for the prioritized five metabolites in the original fecal content samples (control, CD, and UC) and after the addition of G-MBs metabolites at low and high concentrations (see methods). (E–G) TEER (E, F) and CXCL1 secretion (F) measurements in Caco-2 cells treated with only the prioritized five metabolites (G-MBs) or IBD fecal content with and without the addition of five metabolite mix (G-MBs). One-way ANOVA tests were used for the comparison. H, K) Normalized ATP levels are shown after monolayers differentiated colonoid were exposed for 5 h to UC pool #3 and CD pool #2 (H-I) and the indicated IBD pools (UC pool #1, #2 and CD pool #1 and #3, J-K) with and without the five prioritized metabolites (G-MBs) mixture used at two concentrations (G-MBs-low and G-MBs-high; details are provided in the methods and Figure S5E). In I and K, the levels are aggregated and colored by organoids; in H, the levels are summarized in violin plots. t-tests without and with G-MBs supplementation into the UC and CD pools were used for comparison, * *p* < 0.05, **p < 0.01, *** *p* < 0.001, **** *p* < 0.0001, ns *p* > = 0.05.

Previous findings from our group and others^2^^,^^40^ implicated metabolites in the pathogenesis of IBD. Based on these findings and on the observed supernatant origin of the colonoid ATP level reduction (Figure 4D), we focused on characterizing the metabolic contents of the mixed pools and the supernatants. Differential abundance analysis revealed 45 significantly different metabolites between the IBD and healthy samples (FDR ≤ 0.1, Dataset S2, Figure S5A). Among these, 30 metabolites were found at relatively high levels in the control group, and 15 were more abundant in the IBD group. Pathways significantly associated (FDR ≤ 0.1) with the control samples included vitamin B6 metabolism, inositol metabolism, fructose and mannose degradation, and mitochondrial beta-oxidation of long-chain saturated fatty acids. In contrast, the elevated metabolites in IBD patients were associated with arachidonic acid metabolism, tryptophan metabolism, ammonia recycling, and bile acid biosynthesis (Figure S5B). Assuming that metabolites mediate some of these epithelial outcomes, we aimed to identify specific metabolites that can be supplemented into the fecal content of patients with IBD to improve epithelial outcomes. We followed a prioritization approach consisting of three steps: (a) We identified 13 metabolites correlated with improved organoid ATP levels (Figure 4B) and 13 metabolites correlated with Caco-2 TEER (Figure 4C), with an overlap of 8 metabolites between the two groups (Figure 4B). (b) Based on data from a human multi-omics study,^3^ we validated that 12 of these 18 genes were also correlated in vivo with healthier (anti-correlated with disease signals) mucosal epithelial co-expression modules (Figure 5A and S5C). (c) We ensured that the identified metabolites were commercially available for purchase and could be applied within our experimental system (Figure 5B). Through this process, we prioritized five metabolites that were subsequently supplemented into the original fecal content pools: azelate, pyridoxal, fructose-6-phosphate, galactose 1-phosphate, and ribose 5-phosphate, collectively referred to as G-MBs. Four metabolites demonstrated a positive correlation with both TEER (Figure 4C) and ATP levels (Figure 4B, azelate, pyridoxal, fructose-6-phosphate, and galactose 1-phosphate), and we also included ribose phosphate, which was linked with ATP levels in the organoids, together with fructose-6-phosphate, and galactose 1-phosphate, which is linked to ATP production through the TCA cycle (Figure 5C). Finally, we defined and validated the concentrations of the five metabolites by conducting metabolomics analyses and measuring metabolite levels in fecal pool samples without and together with the supplementation of G-MBs (Dataset S2 and Figure 5).

Fecal samples from healthy individuals and those with UC and CD showed variation in the experimental outcomes (Figures 2 and 4A). Among the healthy control samples, control pool #3 had the least impact on colonoid ATP levels and exhibited the weakest effect on CXCL1 and integrity (TEER) in Caco-2 cells. In contrast, UC pools #1 and #3, along with CD pool #2, demonstrated the most pronounced impacts on ATP levels and Caco-2 epithelial integrity, as indicated by TEER. Therefore, we initially tested whether G-MBs can rescue some of the deleterious effects observed with these pools. We wanted to restore epithelial function, which was mostly disrupted by the IBD pools. We noted no toxic effect on Caco-2 and colonoid cells when applying G-MBs in the indicated concentrations (Figure S5D). In Caco-2 cells, adding only G-MBs had, as expected, no effect on TEER, but the addition of G-MBs to the fecal content pools UC p#3 and CD p#2 resulted in improved epithelial integrity. This was demonstrated by a higher TEER when G-MBs were supplemented with the original inflammatory bowel disease (IBD) fecal content compared to TEER with the original IBD pools (Figure 5E). Similar increases in TEER were noted when G-MBs were added to other IBD pools compared to the levels observed with the original indicated IBD pools, which reached significance when G-MBs were added to UC p#1 and CD p#1 pools (Figure 5F). Compared with the original indicated IBD pools, supplementation of UC p#3 and CD p#2 with G-MBs significantly reduced the secretion of CXCL1 from epithelial cells in our model (Figure 5G). In colonoids, adding G-MBs at similar concentrations improved CD p#2 ATP levels. However, a significant increase in the UC p#3 ATP level was only observed at higher concentrations (Figure 5H-I and methods). A significant increase in cellular ATP levels was also noted when adding G-MBs to UC p#1 and CD p#1 (Figure 5). Live imaging using microscopy confirmed the improvement observed with G-MBs in the fecal content pools of UC p#3 and CD p#2, demonstrating enhanced cell morphology and reduced clumping in the monolayered cell culture (Figure S5E). Six metabolites (see extended methods) that were negatively correlated with TEER and ATP (B-MBs) were added to control p#3 at concentrations similar to those observed in the IBD pools (Figure S5F) did not have an effect (Figure S5G-H), as well as adding these metabolites to CD and UC p#1−3 (Figure S5I).

## Discussion

Despite epithelial involvement in the pathogenesis of IBD and gaps in treatment goals with existing immune-directed therapy, epithelial-directed interventions are currently unavailable. Using a preclinical patient-based model, we identified bioactive endogenous metabolite-based interventions that improved IBD epithelial dysfunction in the model. We pooled fecal material from CD, UC, and control subjects to capture patient heterogeneity. Human epithelial cells (Caco-2 cells and patient-derived colonoids) were cultured, with fecal material applied apically to replicate the gut's physiological orientation. Measurable epithelial outputs include epithelial proinflammatory signals, integrity, as measured by TEER, and cellular ATP levels. Overall, we have shown that a. fecal content pools from several independent IBD patients pools disturb epithelial functions significantly more than fecal content from controls, b. the effect on epithelia was linked with fecal metabolite levels and associations were validated using independent previously published in vivo human multi-omics biospecimens cohort^4^ that captured associations of metabolites-epithelial genes network, and c. This process guided the prioritization and supplementation of five metabolites into the IBD fecal content, which reversed the IBD negative effects. The gut host and microbial metabolites include endogenous, potentially bioactive compounds that can influence epithelial metabolism and homeostasis. These compounds are generally regarded as safe and can be easily administered. Our system streamlines a proof-of-concept pipeline for capturing the context of fecal content from IBD patients and controls on human epithelia and for prioritizing epithelial-targeted metabolite interventions, enabling the identification of metabolites that may be used as future interventions, pending further preclinical and clinical validations.

The colonic epithelium is a dynamic metabolic unit that is affected by IBD. Analyses from the UC PROTECT study revealed suppression of epithelial metabolic functions that were validated across cohorts.^4^ We demonstrated reduced activity of the mitochondrial electron transport chain Complex I, an integrated measure of the cell's capacity for ATP production and reduced mitochondrial membrane potential (MMP). These effects may be mediated by the IBD fecal content, which adversely affected the cellular ATP levels of colonic epithelial cells compared to fecal samples from control subjects, as shown here. The observed decrease in metabolite levels in patients with IBD may be attributed to several factors, including differences in microbial composition, as illustrated in Figure 1**.** Additionally, dietary alterations, such as a lower intake of vitamin B6, may explain the reduction in pyridoxal levels. Intrinsic metabolic changes in the patients from whom the samples were collected, especially disruptions in mitochondrial function and carbohydrate metabolism, can impact fecal metabolite profiles. Reduced levels of intermediates, such as fructose-6-phosphate, galactose-1-phosphate, and ribose-5-phosphate, suggest potentially impaired glycolytic and pentose phosphate pathways in epithelial or immune cells, likely reflecting the inflammatory microenvironment. Furthermore, the reduction in fecal azelate is likely influenced by both dietary intake (https://www.hmdb.ca/metabolites/HMDB0000784) and microbial metabolism,^41^ both of which may be altered in individuals with IBD.

In an attempt to overcome part of this metabolic effect, we hypothesized that various host and microbial gut metabolites might contribute small but cumulative positive effects to epithelial ATP levels and epithelial functions. To evaluate the contributions of these metabolites, which may vary in type and quantity across different subjects or pools, we analyzed the relationships between metabolites and the designated epithelial readouts (cellular ATP levels and TEER) using Spearman correlation analysis. Among the functionally prioritized bioactive metabolites, several are directly related to energetic pathways (fructose-6-phosphate, galactose 1-phosphate and ribose 5-phosphate) and are supplemented into the IBD fecal content together with azelate and pyridoxal. Ribose phosphate is an intermediate in the pentose phosphate pathway, which plays a role in RNA/DNA nucleotide synthesis and maintaining redox balance through the production of NADPH, which is essential for cell proliferation and repair.^42^ Galactose-1-phosphate is an intermediate in galactose metabolism, feeds into glycolysis, provides energy (ATP) for cellular functions, and supporting the synthesis of glycoproteins and glycolipids, which are vital for maintaining epithelial cell membrane integrity. Fructose-6-phosphate enters glycolysis, generating ATP and feeding into the hexosamine biosynthetic pathway, producing UDP-*N*-acetylglucosamine, which is essential for glycosylation processes.^42^^,^^43^ Azelate is a naturally occurring dicarboxylic acid recognized for its antioxidant and antibacterial properties, and it is utilized to reduce skin inflammation.^44^^,^^45^ Finally, pyridoxal (vitamin B6) is an active form of vitamin B6, a coenzyme involved in numerous enzymatic reactions, including the synthesis of glutathione, which is crucial for reducing oxidative stress. Subnormal levels of pyridoxal were identified in 29% of IBD patients,^46^ with no significant difference observed between patients with active disease and those in remission. The supplementation of vitamin B6 in IL10 knockout mouse^47^ models improved the levels of molecular and histological markers of inflammation in the colon. These metabolites collectively support gut epithelial cell functions by reducing oxidative stress through NADPH generation (ribose phosphate) and glutathione synthesis (pyridoxal), energy production through glycolytic intermediates (fructose-6-phosphate and galactose-1-phosphate) and ATP production for cellular processes, and reducing inflammation-induced damage (azelate and pyridoxal). The effect noted upon supplementation of these metabolites into the IBD fecal content highlights the therapeutic potential of these endogenous health-associated metabolites for restoring gut epithelial functions.

The more toxic effects observed with the IBD fecal content may be attributable to the IBD microbial composition, which is linked to various virulence factors characteristic of IBD, as noted in other studies.^11^ For example, the presence of *Proteus mirabili*^31^ and *Klebsiella pneumonia*^48-50^ in IBD patients within this cohort was previously associated with urease, hemolysins, and proteases, all of which can disrupt epithelial integrity, promote inflammation, and contribute to the enrichment of ammonia recycling pathways, which are also prominent in the IBD metabolomic profiles analyzed in our study (Figure 5). Additionally, the enrichment of Proteobacteria taxa, including Escherichia coli, is recognized for producing reactive oxygen species (ROS) and lipopolysaccharides (LPS) that can trigger oxidative stress and activate the epithelial immune response.^51^ The upregulated metabolites identified in our models (Dataset S2) included cholate, taurine, and palmitoylcarnitine, all of which could cause membrane damage and mitochondrial dysfunction,^52^^,^^53^ along with arachidonic acid and several fats, such as adrenic acid (all-cis-7,10,13,16-docosatetraenoic acid, an *ω*-6 fatty acid) and mead acid (5(Z),8(Z),11(Z)-eicosatrienoic acid), which may increase inflammation. A recent study examined the effects of fecal water from patients with UC on Caco-2 cells, revealing increased cytotoxicity,^54^ which was also linked to changes in the gut microbiota and the fecal metabolome, which is consistent with our results. However, we expand on these observations, demonstrating the impact of both UC and CD on epithelial functions, including the readouts concerning the epithelial inflammatory response, integrity as measured by TEER, and ATP levels. Furthermore, we utilized ex vivo organoid cultures, which constitute a more physiological system for studying epithelial–microbiota interactions, along with in vitro Caco-2 cells. The use of these complementary systems is exemplified by the effects observed on ATP levels in colonoids but not in Caco-2 cells. This difference likely arises from the cancerous origin of the Caco-2 cells, which tend to exhibit greater metabolic plasticity and potentially higher ATP production. However, despite the absence of effects from the IBD fecal content on ATP in Caco-2 cells, we observed a significant reduction in TEER with independent IBD fecal content compared to control fecal content. Additionally, this diminished integrity has been previously linked with disturbed epithelial metabolic function.^36^^,^^37^ Moreover, we demonstrated higher *TGM2* induction (Figure S1I) with the IBD fecal content in Caco-2 cells, which we have shown to be upregulated in biospecimens from human IBD datasets,^10^^,^^38^and we, along with others, have linked this induction to altered metabolic and mitochondrial functions.^38^ Another novel aspect of our work, compared to previous studies, is that we employ this functional model to direct therapeutic bioactive metabolite interventions as a proof-of-concept to restore epithelial functions as a potential future adjunct intervention.

The fecal pools used in the study were derived from different subjects with varying disease activity levels and microbial compositions. The proportion of active patients in the pool samples, for example, did not seem to explain the effects on epithelia. Notably, we observed more significant effects on epithelial health in CD pool #2 and UC pools #1 and #3. Among the UC and CD patients from pool #1, 50% and 70% were clinically active, respectively. In pool #2, 80% of the UC patients and 20% of the CD patients had clinically active disease, while in pool #3, 80% of the UC patients and 20% of the CD patients were active. These findings suggest that disease activity alone does not fully account for the effects on epithelial health (Table S1−2). Furthermore, medication may have influenced the readout. However, in CD p#1, 6 out of 10 were treatment-naïve and 4 were on anti-TNFs (administered parenterally, not through the gastrointestinal tract). In CD p#2, 3 were treatment-naïve and 2 were anti-TNFs, and in CD p#3, 2 were treatment-naïve and 3 were anti-TNFs. There is a mutual influence between biologics and the microbiota that cannot be ignored; however, since these effects do not occur through the gut, this exposure most likely would not directly affect the gut microbiome or the gut metabolome. Nevertheless, secondary effects may still be possible. The composite gut bacterial indices (Faith diversity index and health index) also did not seem to correlate with epithelial toxicity. CD pool #2, for example, presented greater diversity and health indexes compared to the other CD pools (Figure 1D-E), yet it had a more detrimental effect on epithelial health. Variations in effects were also observed within the non-IBD healthy control pools, with pool #3 demonstrating the least impact on epithelial ATP levels (Figure 5). These differences may be attributed to various factors that cannot be thoroughly assessed within the scope of this study, including dietary exposure, medications (including over-the-counter drugs and supplements), specific bacterial toxins, and other unmeasured metabolites.

Our study has several strengths, we characterized the fecal and metabolite signals in the samples, demonstrating consistency with other IBD cohorts and representation of the associated pathogenic IBD signals. By integrating fecal material with epithelial cells, we examine the complex interactions within this gut microenvironment and compare healthly conditions with those of IBD, with a specific focus on critical aspects of IBD epithelial pathology. The IBD gut microbiome is variable. This is one of the reason we opted to use pools rather than individual samples. While the total number of patients was relatively small, we observed the effect with most outcomes in the three pools, which consisted of different patients. To account for human variability, we pooled fecal material from 10 CD, 10 UC and 10 healthy subjects in p#1 and further validated the findings through two independent pools (#2 and #3), each including samples from five additional subjects. Finally, we take advantage of the variation in metabolite levels in the fecal material and relate these to the epithelial responses. This approach allowed us to prioritize health-associated metabolites that, when added to the IBD fecal content, reduced pro-inflammatory signaling, improved epithelial barrier function, and increased ATP levels in epithelial cells, functions that are typically disrupted in IBD. The limitations of our study include not fully identifying the mediators that influence the detrimental functions of the epithelium. While we used Caco-2 cells, we also incorporated a more physiologic primary colonoids system that better represents human epithelial physiology and variability. We apply heat-killed bacteria rather than live fecal bacteria to account for the variability associated with both host epithelia and fecal content from different individuals and overcome the challenges of culturing aerobic human epithelial cells and anaerobic microbiota. We utilized non-IBD colonoids to isolate the effect of fecal content on epithelia and rather than on IBD epithelia. We did not include animal models, which are typically used for independent in vivo preclinical validation. While animal models are an important part of understanding pathogenesis and potential interventions, these models also have limitations, particularly in regard to capturing the human heterogeneity associated with diet, microbes, and the human host, all of which vary significantly between humans and mice. Our metabolite identification process could be more comprehensive. Using a larger targeted metabolomics library and increasing the number of sample pools could increase statistical power, potentially leading to the discovery of additional beneficial metabolites. While we prioritized and tested five specific metabolites, these may not necessarily be the most effective ones. Our model does not establish that the depletion of these specific metabolites in IBD patients is the direct cause of epithelial dysfunction. Using heat-killed bacteria eliminates the dynamic interaction between live microbes and the host epithelium. However, employing a live bacterial complex consortium in an in vitro system is also challenging, as different bacteria grow at different rates and may not accurately represent the actual gut physiology. Additionally, supplementing metabolites in such a system may not truly reflect their effects within a dynamic gut environment, where live microbes can actively metabolize these compounds. Furthermore, our model does not capture the long-term effects of metabolites on physiological functions, but we aimed to align the concentrations with those seen in the non-IBD fecal content. Nonetheless, our work underscores the potential for identifying and utilizing endogenous gut metabolites that are considered safe for improving epithelial health in IBD patients. Further research is needed to explore the molecular pathways through which beneficial metabolites exert protective effects on epithelial cells.

In conclusion, we describe a patient-based model system to test the effects of disease and healthy fecal contents on epithelial function, prioritize endogenous metabolites, and then test interventions that can improve the epithelial outcomes. This study supports the notion that metabolite-based therapies aimed at supporting epithelial homeostasis can be used as attractive adjunct therapy for IBD.

## Disclosure of potential conflicts of interest

All the authors reported no conflict of interest related to this work.

## Supplementary Material

Supplementary Material DatasetS1_Jan31_2025gg_a.xlsx

Supplementary Material DatasetS2_Apr15_2025_eedits.xlsx

Supplementary Material HL_Apr15_2025_Suppl_File_ref.docx

## Data Availability

Processed 16S data are available as Dataset S1. The 16S amplicon sequencing dataset generated in this study has been deposited in the BioProject under accession code: PRJNA1216163 (https://www.ncbi.nlm.nih.gov/bioproject/PRJNA1216163).

## Abbreviations

ASVs: Amplicon sequence variants
CD: Crohn’s disease
G-MBs: good metabolites
HK: heat-killed bacteria
IBD: inflammatory bowel disease
IQR: interquartile range
LC–MS: liquid chromatography‒mass spectrometry
LPS: lipopolysaccharides
RA: relative abundance
ROS: reactive oxygen species
Sup.: fecal supernatants
TEER: trans-epithelial electrical resistance
UC: ulcerative colitis
UPLC: Ultimate ultrahigh-performance liquid chromatography.

## References

[1] Ning L, Zhou YL, Sun H, Zhang Y, Shen C, Wang Z, Xuan B, Zhao Y, Ma Y, Yan Y, et al. Microbiome and metabolome features in inflammatory bowel disease via multi-omics integration analyses across cohorts. Nat Commun. 2023;14:7135. doi: 10.1038/s41467-023-42788-0. 37932270 PMC10628233

[2] Franzosa EA, Sirota-Madi A, Avila-Pacheco J, Fornelos N, Haiser HJ, Reinker S, Vatanen T, Hall AB, Mallick H, McIver LJ, et al. Gut microbiome structure and metabolic activity in inflammatory bowel disease. Nat Microbiol 2019. 2018;4:293–305. doi: 10.1038/s41564-018-0306-4. PMC6342642 30531976

[3] Braun T, Feng R, Amir A, Levhar N, Shacham H, Mao R, Hadar R, Toren I, Algavi Y, Abu-Saad K, et al. Diet-omics in the study of urban and rural crohn disease evolution (SOURCE) cohort. Nat Commun. 2024;15:3764. doi: 10.1038/s41467-024-48106-6. 38704361 PMC11069498

[4] Haberman Y, Karns R, Dexheimer PJ, Schirmer M, Somekh J, Jurickova I, Braun T, Novak E, Bauman L, Collins MH, et al. Ulcerative colitis mucosal transcriptomes reveal mitochondriopathy and personalized mechanisms underlying disease severity and treatment response. Nat Commun. 2019;10:38. doi: 10.1038/s41467-018-07841-3. 30604764 PMC6318335

[5] Buning C, Geissler N, Prager M, Sturm A, Baumgart DC, Buttner J, Büning C, Büttner J, Bühner S, Haas V, et al. Increased small intestinal permeability in ulcerative colitis: rather genetic than environmental and a risk factor for extensive disease?. Inflamm Bowel Dis. 2012;18:1932–1939. doi: 10.1002/ibd.22909. 22344959

[6] Katinios G, Casado-Bedmar M, Walter SA, Vicario M, Gonzalez-Castro AM, Bednarska O, González-Castro AM, Söderholm JD, Hjortswang H, Keita ÅV. Increased colonic epithelial permeability and mucosal eosinophilia in ulcerative colitis in remission compared with irritable bowel syndrome and health. Inflamm Bowel Dis. 2020;26:974–984. doi: 10.1093/ibd/izz328. 31944236 PMC7301402

[7] Vernia P, Marcheggiano A, Caprilli R, Frieri G, Corrao G, Valpiani D, PAOLO MCD, PAOLUZI P, TORSOLI A. Short-chain fatty acid topical treatment in distal ulcerative colitis. Aliment Pharmacol Ther. 1995;9:309–313. doi: 10.1111/j.1365-2036.1995.tb00386.x. 7654893

[8] Jalili-Firoozinezhad S, Gazzaniga FS, Calamari EL, Camacho DM, Fadel CW, Bein A, Swenor B, Nestor B, Cronce MJ, Tovaglieri A, et al. A complex human gut microbiome cultured in an anaerobic intestine-on-a-chip. Nat Biomed Eng. 2019;3:520–531. doi: 10.1038/s41551-019-0397-0. 31086325 PMC6658209

[9] Valiei A, Aminian-Dehkordi J, Mofrad MRK. Gut-on-a-chip models for dissecting the gut microbiology and physiology. APL Bioeng. 2023;7:011502. doi: 10.1063/5.0126541. 36875738 PMC9977465

[10] Braun T, Sosnovski KE, Amir A, BenShoshan M, VanDussen KL, Karns R, Levhar N, Abbas-Egbariya H, Hadar R, Efroni G, et al. Mucosal transcriptomics highlight lncRNAs implicated in ulcerative colitis, crohn disease, and celiac disease. JCI Insight. 2023;8. doi: 10.1172/jci.insight.170181. PMC10443795 37261910

[11] Haberman Y, Tickle TL, Dexheimer PJ, Kim MO, Tang D, Karns R, Baldassano RN, Noe JD, Rosh J, Markowitz J, et al. Pediatric crohn disease patients exhibit specific ileal transcriptome and microbiome signature. J Clin Invest. 2014;124:3617–3633. doi: 10.1172/JCI75436. 25003194 PMC4109533

[12] Braun T, Di Segni A, BenShoshan M, Neuman S, Levhar N, Bubis M, Picard O, Sosnovski K, Efroni G, Farage Barhom S, et al. Individualized dynamics in the gut microbiota precede Crohn's disease flares. Am J Gastroenterol. 2019;114:1142–1151. doi: 10.14309/ajg.0000000000000136. 30741738

[13] Caporaso JG, Lauber CL, Walters WA, Berg-Lyons D, Huntley J, Fierer N, Owens SM, Betley J, Fraser L, Bauer M, et al. Ultra-high-throughput microbial community analysis on the illumina HiSeq and MiSeq platforms. ISME J. 2012;6:1621–1624. doi: 10.1038/ismej.2012.8. 22402401 PMC3400413

[14] Braun T, Di Segni A, BenShoshan M, Asaf R, Squires JE, Farage Barhom S, Glick Saar E, Cesarkas K, Smollan G, Weiss B, et al. Fecal microbial characterization of hospitalized patients with suspected infectious diarrhea shows significant dysbiosis. Sci Rep. 2017;7:1088. doi: 10.1038/s41598-017-01217-1. 28439072 PMC5430810

[15] Caporaso JG, Kuczynski J, Stombaugh J, Bittinger K, Bushman FD, Costello EK, Fierer N, Peña AG, Goodrich JK, Gordon JI, et al. QIIME allows analysis of high-throughput community sequencing data. Nat Methods. 2010;7:335–336. doi: 10.1038/nmeth.f.303. 20383131 PMC3156573

[16] Bolyen E, Rideout JR, Dillon MR, Bokulich N, Abnet CC, Al-Ghalith GA, Alexander H, Alm EJ, Arumugam M, Asnicar F, et al. Reproducible, interactive, scalable and extensible microbiome data science using QIIME 2. Nat Biotechnol. 2019;37:852–857. doi: 10.1038/s41587-019-0209-9. 31341288 PMC7015180

[17] Amir A, McDonald D, Navas-Molina JA, Kopylova E, Morton JT, Zech Xu Z, Kightley EP, Thompson LR, Hyde ER, Gonzalez A, et al. Deblur rapidly resolves single-nucleotide community sequence patterns. mSystems. 2017;2. doi: 10.1128/mSystems.00191-16. PMC5340863 28289731

[18] McDonald D, Jiang Y, Balaban M, Cantrell K, Zhu Q, Gonzalez A, Morton JT, Nicolaou G, Parks DH, Karst SM, et al. Greengenes2 unifies microbial data in a single reference tree. Nat Biotechnol. 2024;42:715–718. doi: 10.1038/s41587-023-01845-1. 37500913 PMC10818020

[19] Faith DP. Systematics and conservation: on predicting the feature diversity of subsets of taxa. Cladistics. 1992;8:361–373. doi: 10.1111/j.1096-0031.1992.tb00078.x. 34929967

[20] Abbas-Egbariya H, Haberman Y, Braun T, Hadar R, Denson L, Gal-Mor O, Amir A. Meta-analysis defines predominant shared microbial responses in various diseases and a specific inflammatory bowel disease signal. Genome Biol. 2022;23:61. doi: 10.1186/s13059-022-02637-7. 35197084 PMC8867743

[21] Mallick H, Rahnavard A, McIver LJ, Ma S, Zhang Y, Nguyen LH, Tickle TL, Weingart G, Ren B, Schwager EH, et al. Multivariable association discovery in population-scale meta-omics studies. PLoS Comput Biol. 2021;17:e1009442. doi: 10.1371/journal.pcbi.1009442. 34784344 PMC8714082

[22] Mackay GM, Zheng L, van den Broek NJ, Gottlieb E. Analysis of cell metabolism using LC-MS and isotope tracers. Methods Enzymol. Vol. 561 2015; p. 171–196. doi: 10.1016/bs.mie.2015.05.016. 26358905

[23] Pluskal T, Castillo S, Villar-Briones A, Oresic M. MZmine 2: modular framework for processing, visualizing, and analyzing mass spectrometry-based molecular profile data. BMC Bioinform. 2010;11:395. doi: 10.1186/1471-2105-11-395. PMC2918584 20650010

[24] Pang Z, Chong J, Zhou G, de Lima Morais DA, Chang L, Barrette M, Gauthier C, Jacques P, Li S, Xia J. MetaboAnalyst 5.0: narrowing the gap between raw spectra and functional insights. Nucleic Acids Res. 2021;49:W388–W396. doi: 10.1093/nar/gkab382. 34019663 PMC8265181

[25] Lu Y, Pang Z, Xia J. Comprehensive investigation of pathway enrichment methods for functional interpretation of LC-MS global metabolomics data. Brief Bioinform. 2023;24(1). doi: 10.1093/bib/bbac553. PMC9851290 36572652

[26] VanDussen KL, Marinshaw JM, Shaikh N, Miyoshi H, Moon C, Tarr PI, Ciorba MA, Stappenbeck TS. Development of an enhanced human gastrointestinal epithelial culture system to facilitate patient-based assays. Gut. 2015;64:911–920. doi: 10.1136/gutjnl-2013-306651. 25007816 PMC4305344

[27] VanDussen KL, Sonnek NM, Stappenbeck TS. L-WRN conditioned medium for gastrointestinal epithelial stem cell culture shows replicable batch-to-batch activity levels across multiple research teams. Stem Cell Res. 2019;37:101430. doi: 10.1016/j.scr.2019.101430. 30933720 PMC6579736

[28] Co JY, Margalef-Catala M, Monack DM, Amieva MR. Controlling the polarity of human gastrointestinal organoids to investigate epithelial biology and infectious diseases. Nat Protoc. 2021;16:5171–5192. doi: 10.1038/s41596-021-00607-0. 34663962 PMC8841224

[29] Langfelder P, Horvath S. WGCNA: an R package for weighted correlation network analysis. BMC Bioinform. 2008;9:559. doi: 10.1186/1471-2105-9-559. PMC2631488 19114008

[30] Gevers D, Kugathasan S, Denson LA, Vazquez-Baeza Y, Van Treuren W, Ren B, Vázquez-Baeza Y, Schwager E, Knights D, Song SJ, et al. The treatment-naive microbiome in new-onset Crohn's disease. Cell Host Microbe. 2014;15:382–392. doi: 10.1016/j.chom.2014.02.005. 24629344 PMC4059512

[31] Zhang J, Hoedt EC, Liu Q, Berendsen E, Teh JJ, Hamilton A, O’ Brien AW, Ching JY, Wei H, Yang K, et al. Elucidation of proteus mirabilis as a key bacterium in Crohn's disease inflammation. Gastroenterology. 2021;160:317–330 e11. doi: 10.1053/j.gastro.2020.09.036. 33011176

[32] Cao Y, Oh J, Xue M, Huh WJ, Wang J, Gonzalez-Hernandez JA, Rice TA, Martin AL, Song D, Crawford JM, et al. Commensal microbiota from patients with inflammatory bowel disease produce genotoxic metabolites. Science. 2022;378:eabm3233. doi: 10.1126/science.abm3233. 36302024 PMC9993714

[33] Friedrich M, Pohin M, Jackson MA, Korsunsky I, Bullers SJ, Rue-Albrecht K, Christoforidou Z, Sathananthan D, Thomas T, Ravindran R, et al. IL-1-driven stromal-neutrophil interactions define a subset of patients with inflammatory bowel disease that does not respond to therapies. Nat Med. 2021;27:1970–1981. doi: 10.1038/s41591-021-01520-5. 34675383 PMC8604730

[34] Li J, Simmons AJ, Hawkins CV, Chiron S, Ramirez-Solano MA, Tasneem N, Kaur H, Xu Y, Revetta F, Vega PN, et al. Identification and multimodal characterization of a specialized epithelial cell type associated with Crohn's disease. Nat Commun. 2024;15:7204. doi: 10.1038/s41467-024-51580-7. 39169060 PMC11339313

[35] Mumy KL, McCormick BA. The role of neutrophils in the event of intestinal inflammation. Curr Opin Pharmacol. 2009;9:697–701. doi: 10.1016/j.coph.2009.10.004. 19854677 PMC2798135

[36] Ling C, Versloot CJ, Arvidsson Kvissberg ME, Hu G, Swain N, Horcas-Nieto JM, Miraglia E, Thind MK, Farooqui A, Gerding A, et al. Rebalancing of mitochondrial homeostasis through an NAD(+)-SIRT1 pathway preserves intestinal barrier function in severe malnutrition. EBioMedicine. 2023;96:104809. doi: 10.1016/j.ebiom.2023.104809. 37738832 PMC10520344

[37] JanssenDuijghuijsen LM, Grefte S, de Boer VCJ, Zeper L, van Dartel DAM, van der Stelt I, Bekkenkamp-Grovenstein M, van Norren K, Wichers HJ, Keijer J. Mitochondrial ATP depletion disrupts Caco-2 monolayer integrity and internalizes claudin 7. Front Physiol. 2017;8:794. doi: 10.3389/fphys.2017.00794. 29075202 PMC5641570

[38] Sosnovski KE, Braun T, Amir A, Moshel D, BenShoshan M, VanDussen KL, Levhar N, Abbas-Egbariya H, Beider K, Ben-Yishay R, et al. GATA6-AS1 regulates intestinal epithelial mitochondrial functions, and its reduced expression is linked to intestinal inflammation and less favorable disease course in ulcerative colitis (UC). J Crohns Colitis. 2023;17:960–971. doi: 10.1093/ecco-jcc/jjad006. 36655602 PMC10274303

[39] Grosheva I, Zheng D, Levy M, Polansky O, Lichtenstein A, Golani O, Dori-Bachash M, Moresi C, Shapiro H, Del Mare-Roumani S, et al. High-throughput screen identifies host and microbiota regulators of intestinal barrier function. Gastroenterology. 2020;159:1807–1823. doi: 10.1053/j.gastro.2020.07.003. 32653496

[40] Schirmer M, Strazar M, Avila-Pacheco J, Rojas-Tapias DF, Brown EM, Temple E, Stražar M, Deik A, Bullock K, Jeanfavre S, et al. Linking microbial genes to plasma and stool metabolites uncovers host-microbial interactions underlying ulcerative colitis disease course. Cell Host Microbe. 2024;32:209–226 e7. doi: 10.1016/j.chom.2023.12.013. 38215740 PMC10923022

[41] Yang J, Liu S, Zhao Q, Li X, Jiang K. Gut microbiota-related metabolite alpha-linolenic acid mitigates intestinal inflammation induced by oral infection with toxoplasma gondii. Microbiome. 2023;11:273. doi: 10.1186/s40168-023-01681-0. 38087373 PMC10714487

[42] TeSlaa T, Ralser M, Fan J, Rabinowitz JD. The pentose phosphate pathway in health and disease. Nat Metab. 2023;5:1275–1289. doi: 10.1038/s42255-023-00863-2. 37612403 PMC11251397

[43] Kim YH, Nakayama T, Nayak J. Glycolysis and the hexosamine biosynthetic pathway as novel targets for upper and lower airway inflammation. Allergy Asthma Immunol Res. 2018;10:6–11. doi: 10.4168/aair.2018.10.1.6. 29178672 PMC5705485

[44] Zhang D, Luo Z, Jin Y, Chen Y, Yang T, Yang Q, Wu B, Shang Y, Liu X, Wei Y, et al. Azelaic acid exerts antileukemia effects against acute myeloid leukemia by regulating the Prdxs/ROS signaling pathway. Oxid Med Cell Longev. 2020;2020:1295984. doi: 10.1155/2020/1295984. 33425206 PMC7775164

[45] Jones DA. Rosacea, reactive oxygen species, and azelaic acid. J Clin Aesthet Dermatol. 2009;2:26–30. PMC2958186 20967185

[46] Vagianos K, Bector S, McConnell J, Bernstein CN. Nutrition assessment of patients with inflammatory bowel disease. JPEN J Parenter Enteral Nutr. 2007;31:311–319. doi: 10.1177/0148607107031004311. 17595441

[47] Selhub J, Byun A, Liu Z, Mason JB, Bronson RT, Crott JW. Dietary vitamin B6 intake modulates colonic inflammation in the IL10-/- model of inflammatory bowel disease. J Nutr Biochem. 2013;24:2138–2143. doi: 10.1016/j.jnutbio.2013.08.005. 24183308 PMC4199223

[48] Abbas SMS, Yousef HA, Saleh N. MM. Crippling of klebsiella pneumoniae virulence by metformin, N-acetylcysteine and secnidazole. BMC Microbiol. 2023;23:229. doi: 10.1186/s12866-023-02969-9. 37608306 PMC10464179

[49] Federici S, Kredo-Russo S, Valdes-Mas R, Kviatcovsky D, Weinstock E, Matiuhin Y, Valdés-Mas R, Silberberg Y, Atarashi K, Furuichi M, et al. Targeted suppression of human IBD-associated gut microbiota commensals by phage consortia for treatment of intestinal inflammation. Cell. 2022;185:2879–2898 e24. doi: 10.1016/j.cell.2022.07.003. 35931020

[50] Riwu KHP, Effendi MH, Rantam FA, Khairullah AR, Widodo A. A review: virulence factors of klebsiella pneumonia as emerging infection on the food chain. Vet World. 2022;15:2172–2179. doi: 10.14202/vetworld.2022.2172-2179. 36341059 PMC9631384

[51] Mukhopadhya I, Hansen R, El-Omar EM, Hold GL. IBD-what role do proteobacteria play?. Nat Rev Gastroenterol Hepatol. 2012;9:219–230. doi: 10.1038/nrgastro.2012.14. 22349170

[52] Smith SA, Ogawa SA, Chau L, Whelan KA, Hamilton KE, Chen J, Tan L, Keilbaugh S, Fogt F, Bewtra M, et al. Mitochondrial dysfunction in inflammatory bowel disease alters intestinal epithelial metabolism of hepatic acylcarnitines. J Clin Invest. 2021;131. doi: 10.1172/JCI133371. PMC7773399 33141762

[53] Vich Vila A, Hu S, Andreu-Sanchez S, Collij V, Jansen BH, Augustijn HE, Andreu-Sánchez S, Bolte LA, Ruigrok RAAA, Abu-Ali G, et al. Faecal metabolome and its determinants in inflammatory bowel disease. Gut. 2023;72:1472–1485. doi: 10.1136/gutjnl-2022-328048. 36958817 PMC10359577

[54] Poppe J, Boesmans L, Vieira-Silva S, Deroover L, Tito R, Vandeputte D, Vandermeulen G, De Preter V, Raes J, Vermeire S, et al. Differential contributions of the gut microbiota and metabolome to pathomechanisms in ulcerative colitis: an in vitro analysis. Gut Microbes. 2024;16:2424913. doi: 10.1080/19490976.2024.2424913. 39535140 PMC11562902

