# Supplementary material for: Supplementation with endogenous healthy gut metabolites reverses the disruptions of in vitro and ex vivo epithelial functions induced by fecal content from IBD patients
Source: Gut Microbes. 2025 Dec 15;17(1):2597568. doi: 10.1080/19490976.2025.2597568 (PMC12710890; doi:10.1080/19490976.2025.2597568)
Supplement: Supplementary Material — HL_Apr15_2025_Suppl_File_ref.docx [file KGMI_A_2597568_SM6605.docx]

**Supplementation with endogenous healthy gut metabolites reverses the disruptions of in vitro and ex vivo epithelial functions induced by fecal content from IBD patients**

Haya Abbas-Egbariya*^1,2^, Lubna Elwahidi*^1,2^, David Jessula Levy^1^, Tzipi Braun^1^, Nina Levhar^1,2^, Rotem Hadar^1^, Gilat Efroni^1^, Maya Granot^1,2^, Yael Leichtmann-Bardogoo^5^, Ben M. Maoz^5,6,7,8^, Batia Weiss^1,2^, Ohad Gal-Mor^2,3,4^, Bella Agranovich^9,10^, Ifat Abramovich^9,10^, Lee Denson^11^, Shomron Ben-Horin^1,2^, Kelli L. VanDussen^11^, Amnon Amir^1^^, Yael Haberman^1, 2, 11^^

^1^Sheba Medical Center, Tel-Hashomer, affiliated with the School of Medicine, Faculty of Medical and Health Sciences, Tel-Aviv University, Tel Aviv, Israel.

^2^Gray Faculty of Medicine, Faculty of Medical and Health Sciences, Tel-Aviv University, Tel-Aviv, Israel

^3^The Infectious Diseases Research Laboratory, Sheba Medical Center, Tel-Hashomer, Israel

^4^Department of Clinical Microbiology and Immunology, Tel Aviv University, Tel Aviv, Israel

^5^Department of Biomedical Engineering, and Sagol School of Neuroscience at Tel Aviv University.

^6^Sagol School of Neuroscience Tel-Aviv University, Tel-Aviv, Israel

^7^ The Center for Nanoscience and Nanotechnology, Tel Aviv University, Tel Aviv 69978, Israel

^8^The George S. Wise Faculty of Life Sciences, Tel Aviv University, Tel Aviv 69978, Israel

^9^Laura and Isaac Perlmutter Metabolomics Center, Technion-Israel Institute of Technology, Bat Galim, Haifa, Israel

^10^The Ruth and Bruce Rappaport Faculty of Medicine, Technion-Israel Institute of Technology, Bat Galim, Haifa, Israel.

^11^Cincinnati Children’s Hospital Medical Center, Department of Pediatrics, University of Cincinnati College of Medicine, Cincinnati, OH, USA.

*Equal contribution

^Equal contribution

**Supporting information**

**Supplementary PDF file include:**

1. **Supplementary Figures S1-S4**
2. **Table S1:** Characteristics of ulcerative colitis (UC), Crohn's disease (CD), and control subjects with fecal samples included in pool #1.
3. **Table S2:** Characteristics of ulcerative colitis (UC), Crohn's disease (CD), and control subjects with fecal samples included in pools #2 & #3.
4. **Table S3:** Characteristics of subjects from which we generated colon-derived organoid culture.
5. **Extended methods**

**Dataset S1 (separate spreadsheet):** 16S metadata, amplicon sequence variants (ASVs) bacterial sequences, and processed data.

**Dataset S2 (separate spreadsheet):** Metabolites prioritization.

**
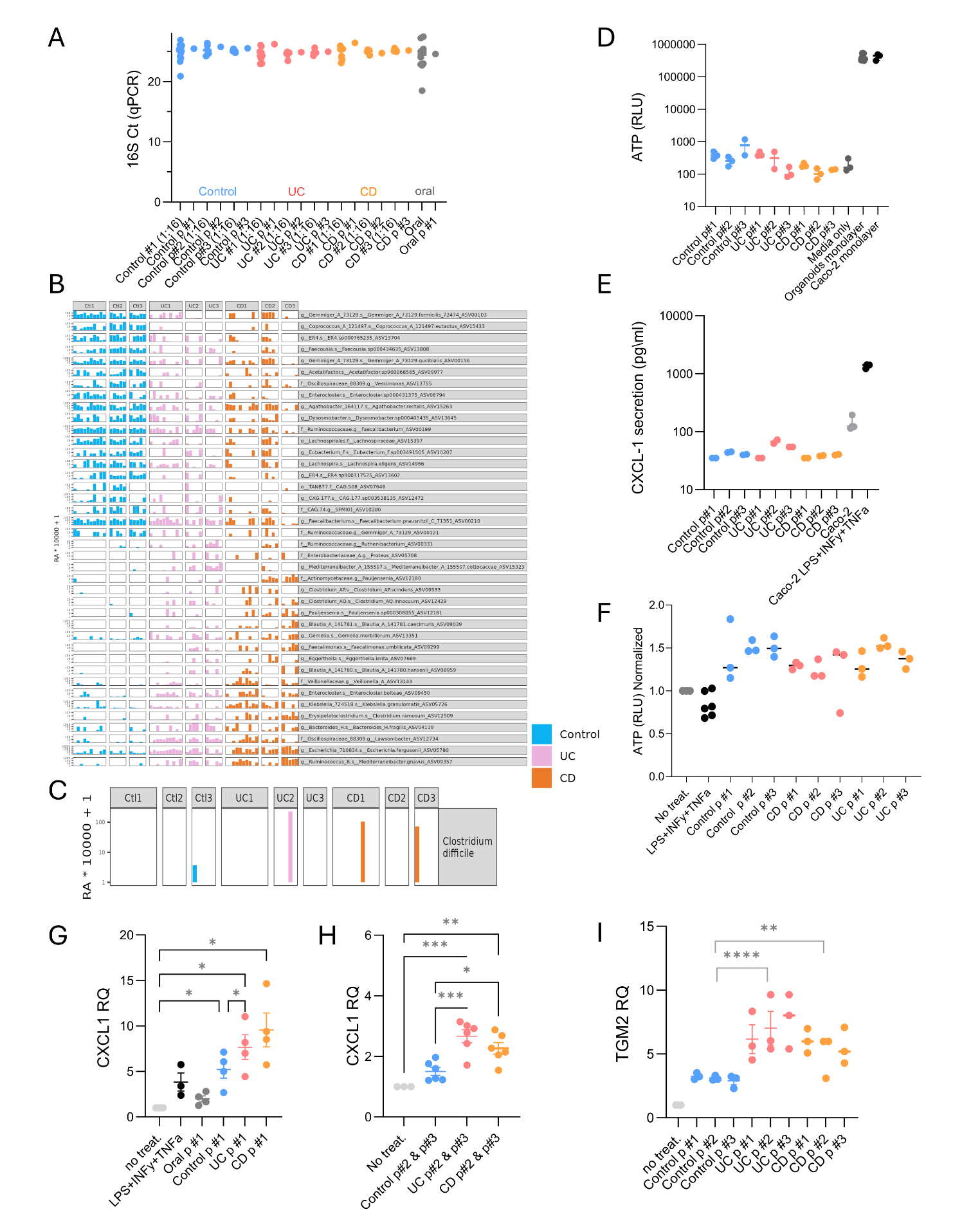
Figure S1. Additional characterization of heat-inactivated fecal and pooled samples A.** Quantifying bacterial DNA by qPCR represents Ct values after sample processing. Fecal content samples that were used to generate pools #1, #2, and #3 as part of the preparation were diluted 1:10, and were additionally diluted (1:16) before qPCR analysis. **B**. Normalized relative abundance (RA × 10,000 + 1) of top 20 ASVs higher or lower in IBD vs. controls that resulted from Maaslin2 differential abundance analyses, controlling for age and gender (**full list in Dataset S1,** where each ASV number is related to a specific sequence). Results are grouped into three diagnostic categories: controls (Ctl p#1-3), ulcerative colitis (UC p#1-3), and Crohn’s disease (CD p#1-3). **C.** Normalized relative abundance (RA × 10,000 + 1) of ASV corresponding to *Clostridium difficile* (see methods for the indicated sequence). **D.** ATP levels in fecal content pools, the culture medium of untreated colonoids monolayer, and Caco-2 using the ATP-Glo assay, showing relatively low ATP levels in the pools. **E**. Quantification of CXCL-1 levels in fecal content pools p#1-3, untreated Caco-2 monolayers, and Caco-2 monolayers stimulated with inflammatory triggers (LPS + IFNγ + TNFα). **F.** Caco-2 cell ATP levels were assessed 24 hours post-exposure using the ATP-Glo Cell ATP levels Luminescence Kit, with values normalized to the untreated control group. **G-H**. *CXCL1* mRNA levels (RQ normalized to GAPDH) are shown for p#1 (G) and p#2-3 (H). **I.** *TGM2* mRNA levels (RQ normalized to GAPDH) are shown for the non-IBD and IBD fecal content pools. One-way ANOVA with Šídák's multiple comparisons test was used for comparison. * p < 0.05, **p < 0.01, ***p < 0.001.

**
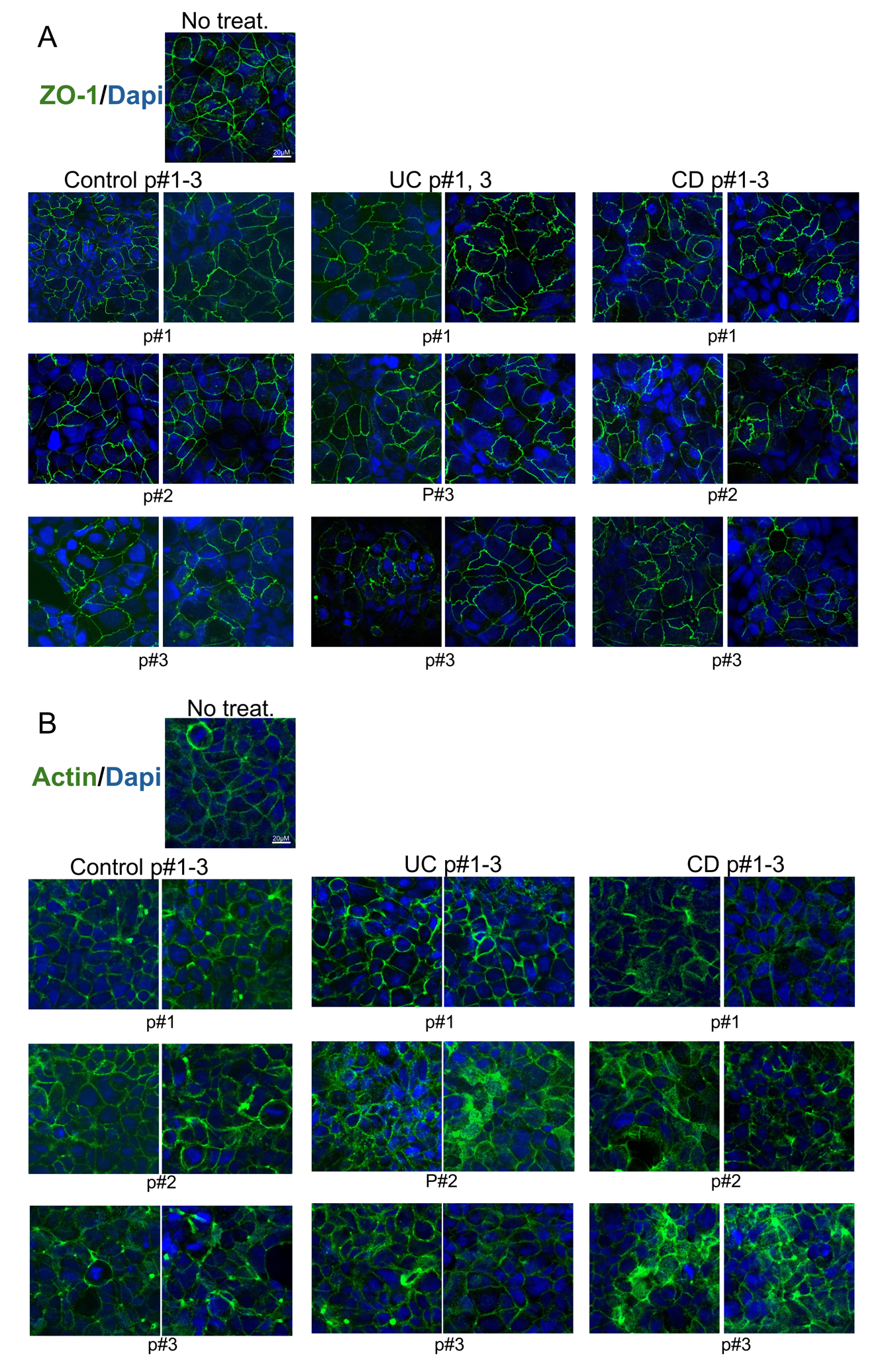
**

**Figure S2. Actin and ZO1 staining upon Caco-2 treatment with fecal content.** Representative confocal microscopy images of monolayer Caco-2 stained for (ZO-1, green, **A**) and nuclei (DAPI, blue), and for actin filaments (phalloidin, green, **B**), and nuclei (DAPI, blue) after 24 hours incubation with fecal pools as indicated or left untreated. All images were taken in one visit with the same parameters and size. Bars are shown in untreated but apply to all images.

**
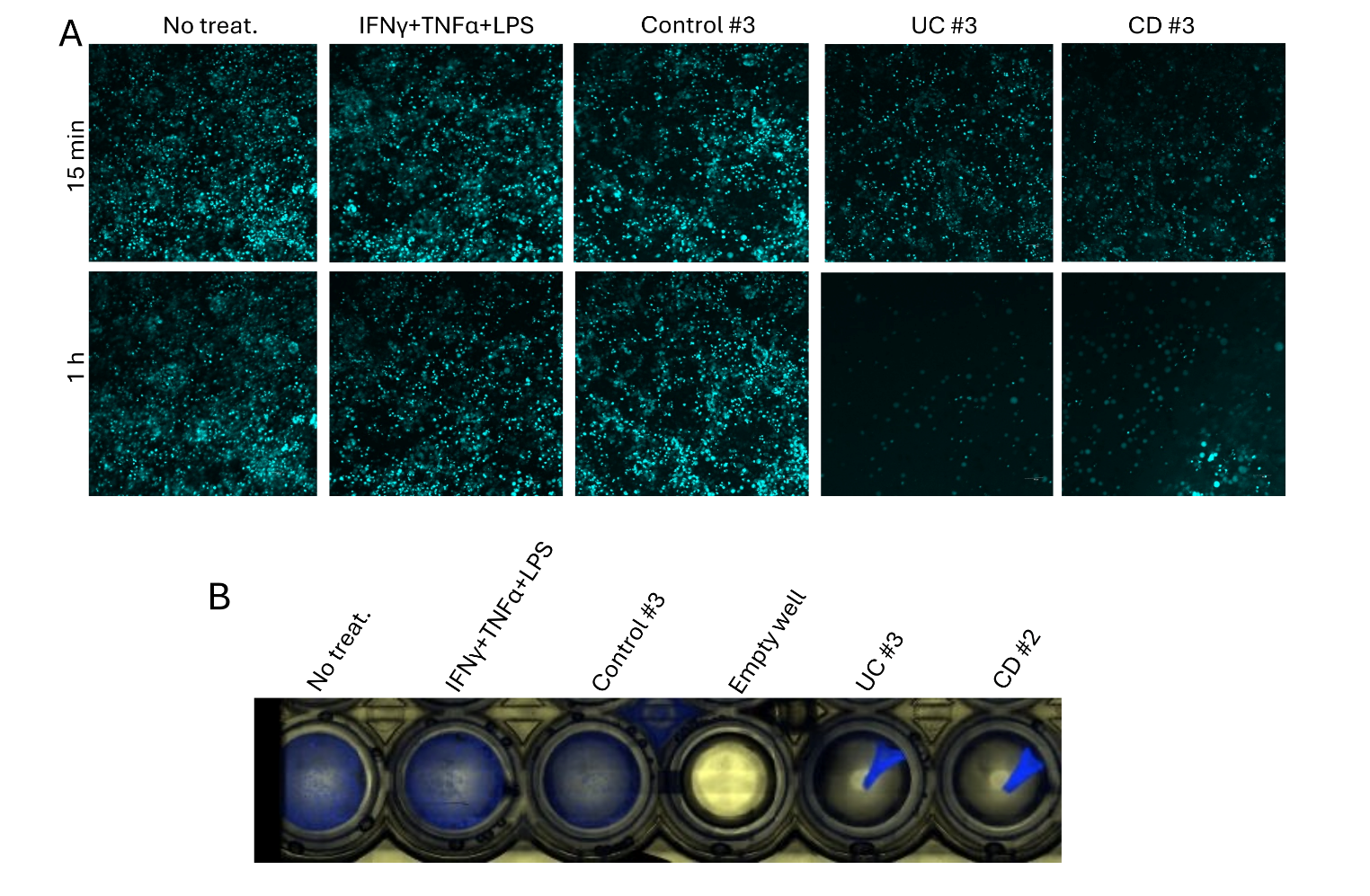
Figure S3. Fecal heat-inactivated pools from IBD patients induce notable changes in colonoid monolayer morphology. A**. Live confocal imaging of colonoid monolayer, differentiated for 72 hours. Hoechst stain was added just before intervention to all wells. The upper row shows the colonoid morphology after 15 minutes of treatment, and the lower row displays the same well after 1 hour of treatment. **B**. Fluorescent imaging of the same control colonoid as in (A), stained with Hoechst and imaged at 4× magnification using a fluorescent microscope, after 2 hours of treatment.

**
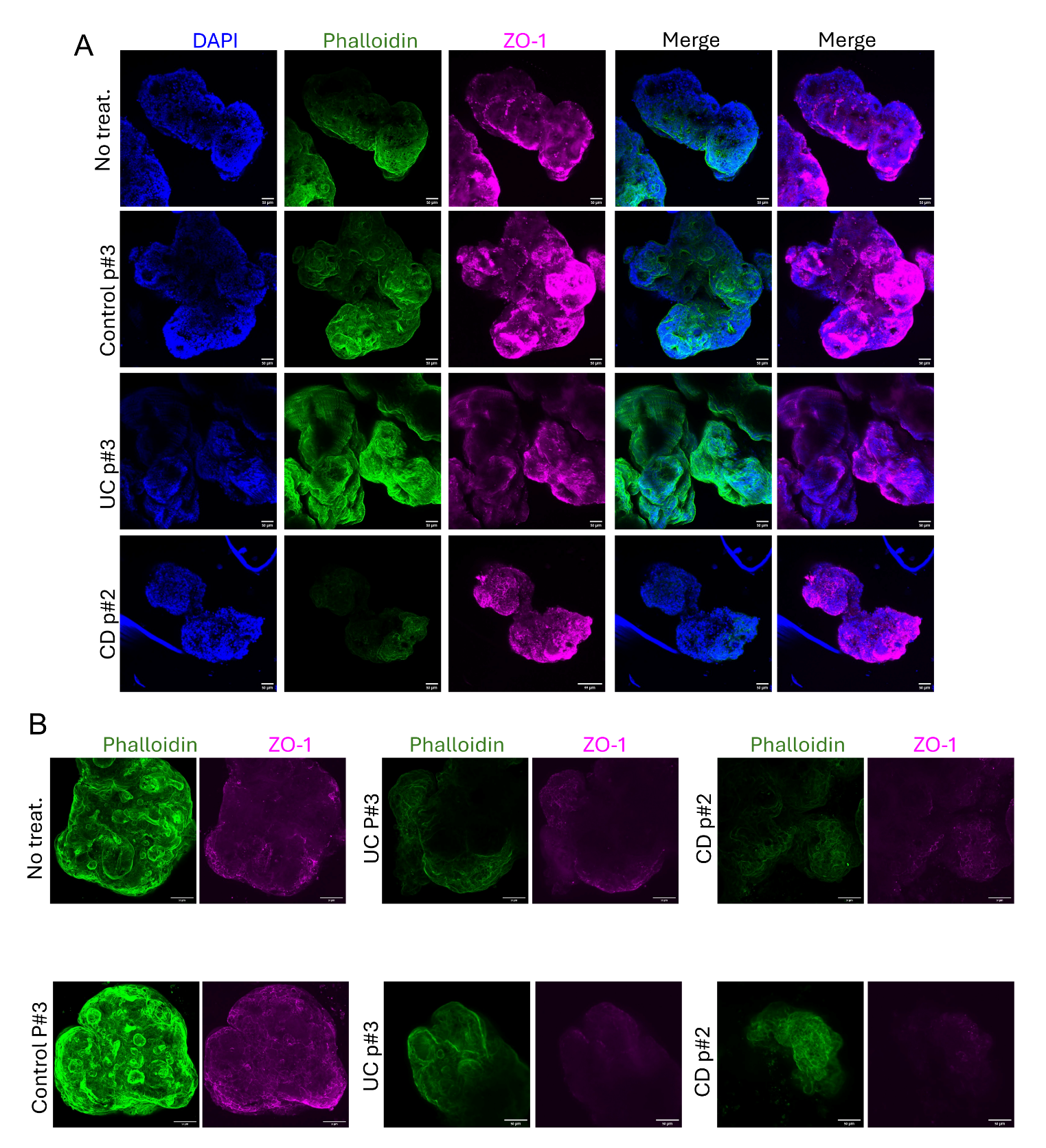
Figure S4. Actin and ZO1 staining upon treatment of apical-out 3D colonoids with fecal contents.** Representative confocal microscopy images (20× magnification) of 3D apical-out colonoids stained for nuclei (DAPI, blue), actin filaments (phalloidin, green), and tight junctions (ZO-1, purple) after 5 hours incubation with fecal pools: control p#3, UC p#3, CD #2, or untreated colonoids. All images in A and inn B were taken in one visit with the same parameters and size. Bars represent 50µM.

**
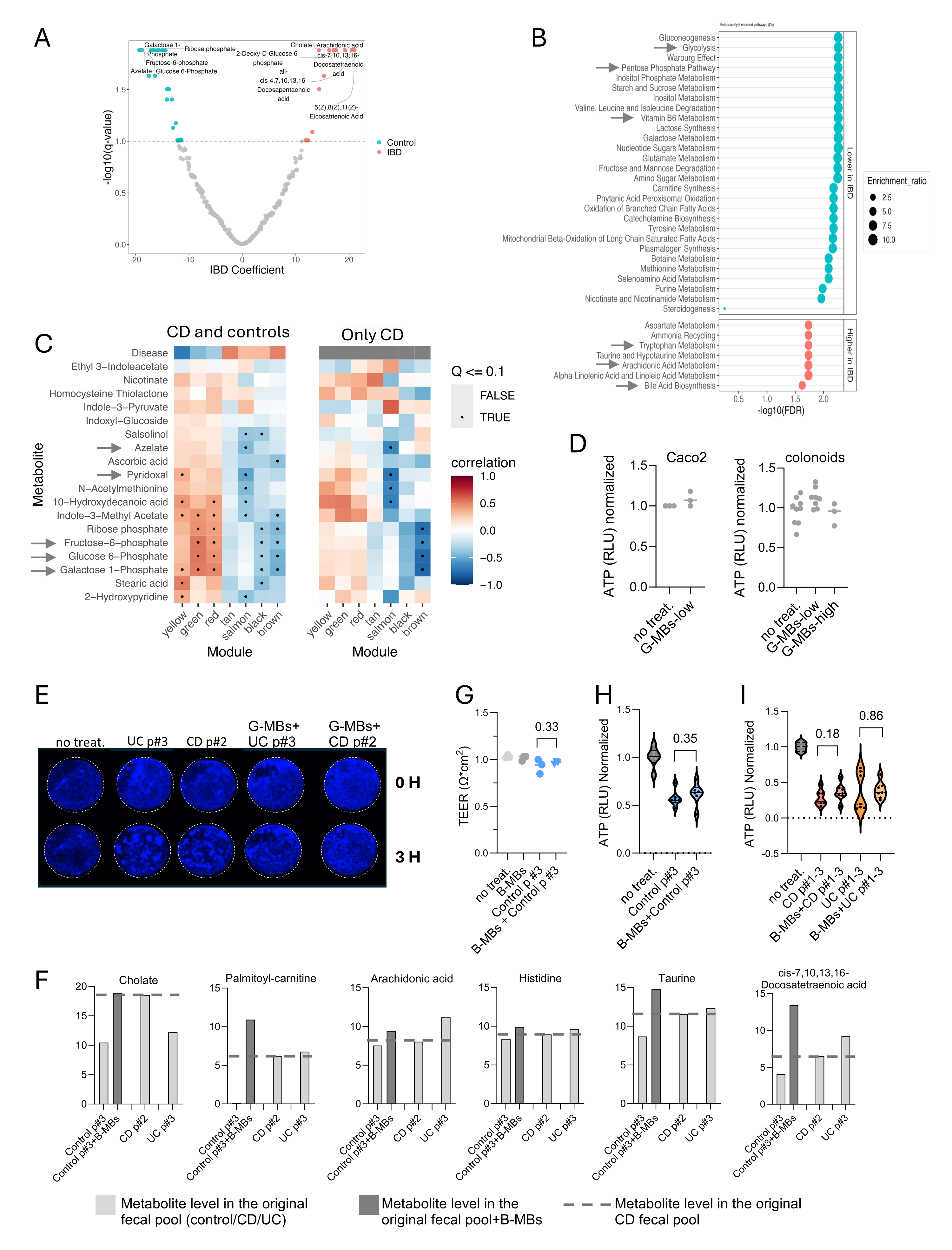
**

**Figure S5. Prioritization and Selection of Metabolites that were supplemented in the fecal content experiments. A**. Volcano plot of 45 differentially abundant metabolites between IBD and control samples (Calour dsFDR≤ 0.1). Gray dots represent non-significant metabolites. Metabolites higher in IBD are colored in red (positive coefficients) and higher in controls in blue (negative coefficients). The dotted line represents FDR = 0.1. **B.** Metabolites functional enrichment analysis (MetaboAnalyst) on the differentially abundant metabolites presented in (A) shows the enrichment ratio and FDR for the top pathways (enrichment ratio ≥ 7, FDR ≤ 0.1). **C**. Expansion of main Fig. 5A. In-vivo cross-validation between metabolite levels and mucosal transcriptomics epithelial modules based on a previously published multi-omics dataset^1^, focusing on 18 metabolites. Left: analysis of control and CD patients. Right: Analysis within CD patients only. The module key is provided in Figure 5. Color represents the correlation coefficient for each comparison. Correlations with Benjamini-Hochberg FDR values ≤ 0.1 are marked with black dots. **D.** Metabolites by themselves do not affect ATP levels**. E**. Imaging of colonoids stained with Hoechst using a fluorescent microscope at 4× magnification. Images were captured at baseline (0 hours) and after 3 hours of exposure to the indicated fecal supernatant-only interventions without and with G-MBs. F. Bar graph showing normalized metabolite levels (Total Sum Scaling (TSS) normalization) for the prioritized six metabolites in the original fecal content samples (control, CD, and UC) and after the addition of B-MBs metabolites. **G-H**. TEER Caco-2 cells (G) and ATP levels in colonoids (G) treated with control fecal content with and without the addition of a six-metabolite mix (B-MBs). **I.** ATP levels in colonoids treated with IBD fecal contents with and without the addition of a six-metabolite mix (B-MBs). Two-tailed paired t-tests were used for the comparison.

| **Table S1:**  **Characteristics of ulcerative colitis (UC), Crohn's disease (CD), and control subjects with fecal samples included in pool #1.** | | | | | | | | | | |
| --- | --- | --- | --- | --- | --- | --- | --- | --- | --- | --- |
| **Sample** | **Dx** | **age** | **Sex** | **BMI** | **Smoking** | **PGA** | **Treatment** | **UC-loc** | **CRP** | **calpro** |
| p#1.1 | UC | 23 | M | 28.3 | uk | Active | *None | E3 | 15 | >1000 |
| p#1.2 | UC | 19 | M | 25.1 | uk | Active | *None | E3 | 4.9 | 969 |
| p#1.3 | UC | 18 | F | 29 | no | Remission | anti-TNF+IM | E3 | 1.4 | 359 |
| p#1.4 | UC | 16 | M | 25 | no | Remission | anti-TNF | E3 | 0.2 | 547 |
| p#1.5 | UC | 12 | M | 18.3 | no | Remission | anti-TNF+IM | E3 | 0.55 | 968 |
| p#1.6 | UC | 19 | M | 20 | no | Active | anti-TNF+IM | E2 | 25 | 30 |
| p#1.7 | UC | 14 | F | 23 | no | Remission | anti-integrin | E3 | 0.5 | 100 |
| p#1.8 | UC | 20 | M | 19.4 | no | Remission | anti-TNF | E3 | 0.5 | 220 |
| p#1.9 | UC | 12 | M | 19.9 | no | Active | anti-TNF | E3 | 0.5 | 420 |
| p#1.10 | UC | 19 | F | 19 | no | Active | anti-integrin | E3 | 30 | uk |
| Summary pool #1 UC: 10 patients with median age 19years, 70% males, 50% with active disease, median fecal calprotectin 420 | | | | | | | | | | |
| **Sample** | **Dx** | **age** | **Sex** | **BMI** | **Smoking** | **PGA** | **Treatment** | **CD-loc** | **CRP** | **calpro** |
| p#1.11 | CD | 23 | M | 18.3 | yes | Active | *None | L1 | 6.8 | >1000 |
| p#1.12 | CD | 25 | M | 20.7 | no | Active | *None | L1 | 33.2 | >1000 |
| p#1.13 | CD | 32 | M | uk | uk | Active | *None | L2 | 4.72 | 975 |
| p#1.14 | CD | 30 | M | 34.7 | no | Active | *None | L3 | 20.6 | >1000 |
| p#1.15 | CD | 19 | F | 23.4 | no | Active | anti-TNF | L3 | 9.35 | >1000 |
| p#1.16 | CD | 42 | F | 22.7 | no | Active | *None | L3 | 65 | >1000 |
| p#1.17 | CD | 22 | M | 18.6 | no | Remission | anti-TNF | L1 | 3.87 | 78 |
| p#1.18 | CD | 20 | F | 19.3 | no | Remission | anti-TNF | L3 | 19.9 | >1000 |
| p#1.19 | CD | 32 | F | 19.2 | uk | Remission | anti-TNF | L1 | 1.27 | 30 |
| p#1.20 | CD | 21 | M | 23.5 | no | Active | *None | L3 | 11.2 | 663 |
| Summary pool #1 CD: 10 patients with median age 24years, 60% males, 70% with active disease, median fecal calprotectin 1000 | | | | | | | | | | |
| **Sample** | **Dx** | **age** | **Sex** | **BMI** | **Smoking** |  |  |  |  |  |
| p#1.21 | control | 46 | F | 20.8 | no |  |  |  |  |  |
| p#1.22 | control | 26 | F | 24.9 | uk |  |  |  |  |  |
| p#1.23 | control | 30 | F | 19 | no |  |  |  |  |  |
| p#1.24 | control | 19 | M | 30.4 | uk |  |  |  |  |  |
| p#1.25 | control | 26 | M | 23.8 | no |  |  |  |  |  |
| p#1.26 | control | 28 | F | 18 | uk |  |  |  |  |  |
| p#1.27 | control | 18 | F | 21 | yes |  |  |  |  |  |
| p#1.28 | control | 34 | M | 22.2 | uk |  |  |  |  |  |
| p#1.29 | control | 24 | M | uk | no |  |  |  |  |  |
| p#1.30 | control | 24 | M | 25.3 | no |  |  |  |  |  |
| Summary pool #1 healthy controls: 10 subjects with median age 26years, 50% males | | | | | | | | | | |
| *None; treatment naïve newly diagnosed, uk; unknown. >1000, kit linear range. | | | | | | | | | | |

| **Table S2:**  **Characteristics of ulcerative colitis (UC), Crohn's disease (CD), and control subjects with fecal samples included in pools #2 & #3.** | | | | | | | | | | | |
| --- | --- | --- | --- | --- | --- | --- | --- | --- | --- | --- | --- |
| **Sample** | **Dx** | **age** | **Sex** | **BMI** | **Smoking** | **PGA** | **Treatment** | **UC-loc** | **CRP** | **calpro** |  |
| p#2.1 | UC | 17 | F | 23.3 | no | Remission | IM 5ASA | E3 | 3 | 242 |  |
| p#2.2 | UC | 30 | M | 18.1 | yes | Active | anti-TNF  budesonide | E3 | 4.3 | >1000 |  |
| p#2.3 | UC | 26 | F | 25.3 | no | Active | anti-integrin | E3 | 8.7 | >1000 |  |
| p#2.4 | UC | 39 | M | 22.8 | no | Active | 5ASA | E2 | 2.1 | >1000 |  |
| p#2.5 | UC | 50 | M | 34.9 | no | Active | 5ASA budesonide | E2 | 1.5 | 168 |  |
| Summary pool #2 UC: 5 patients with median age 30years, 60% males, 80% with active disease, median fecal calprotectin 1000 | | | | | | | | | | | |
| p#3.1 | UC | 16 | M | 17 | no | Remission | anti-integrin | E3 | 4.8 | 75 |  |
| p#3.2 | UC | 37 | M | 22.7 | no | Active | 5ASA | E2 | 2.0 | >1000 |  |
| p#3.3 | UC | 28 | F | 24.2 | no | Active | 5ASA | E2 | 12.7 | >1000 |  |
| p#3.4 | UC | 35 | F | 17.9 | no | Active | 5ASA | E2 | 42 | >1000 |  |
| p#3.5 | UC | 23 | F | 23.5 | no | Active | 5ASA | E3 | 5.9 | >1000 |  |
| Summary pool #3 UC: 5 patients with median age 32years, 60% males, 80% with active disease, median fecal calprotectin 1000 | | | | | | | | | | | |
| **Sample** | **Dx** | **age** | **Sex** | **BMI** | **Smoking** | **PGA** | **Treatment** | **CD-loc** | **CRP** | **calpro** |  |
| p#2.6 | CD | 50 | M | 23.9 | no | Active | anti-TNF | L2 | 6.24 | 558 |  |
| p#2.7 | CD | 35 | M | 36 | uk | Remission | none | L1 | 11.1 | 131 |  |
| p#2.8 | CD | 31 | M | 26 | no | Remission | none | L1 | uk | 259 |  |
| p#2.9 | CD | 25 | F | 20 | no | Remission | none | L1 | 0.71 | 30 |  |
| p#2.10 | CD | 30 | M | 23.5 | no | Remission | anti-TNF | L1 | 1.55 | 30 |  |
| Summary pool #2 CD: 5 patients with median age 31years, 60% males, 20% with active disease, median fecal calprotectin 131 | | | | | | | | | | | |
| p#3.6 | CD | 19 | F | 20.1 | no | Remission | none | L3 | 1.06 | 138 |  |
| p#3.7 | CD | 25 | F | 19.8 | no | Remission | anti-TNF | L1 | 0.57 | 30 |  |
| p#3.8 | CD | 45 | M | 22.7 | no | Remission | anti-TNF | L1 | 0.25 | 30 |  |
| p#3.9 | CD | 59 | M | 20.5 | uk | Active | none | L1 | 8.87 | 344 |  |
| p#3.10 | CD | 22 | F | 27 | no | Remission | anti-TNF | L1 | 5.47 | 401 |  |
| Summary pool #3 CD: 5 patients with median age 35years, 60% males, 20% with active disease, median fecal calprotectin 84 | | | | | | | | | | | |
| **Sample** | **Dx** | **age** | **Sex** | **BMI** | **Smoking** |  |  |  |  |  |  |
| p#2.11 | control | 59 | M | 29.4 | yes |  |  |  |  |  |  |
| p#2.12 | control | 46 | M | na | no |  |  |  |  |  |  |
| p#2.13 | control | 39 | M | 24.3 | no |  |  |  |  |  |  |
| p#2.14 | control | 55 | F | 28.3 | no |  |  |  |  |  |  |
| p#2.15 | control | 60 | M | 24.5 | no |  |  |  |  |  |  |
| Summary pool #2 healthy controls: 5 subjects with median age 55years, 80% males | | | | | | | | | | | |
| p#3.11 | control | 60 | F | 19 | no |  |  |  |  |  |  |
| p#3.12 | control | 45 | F | 20 | no |  |  |  |  |  |  |
| p#3.13 | control | 46 | M | uk | no |  |  |  |  |  |  |
| p#3.14 | control | 6 | M | 23 | no |  |  |  |  |  |  |
| p#3.15 | control | 45 | M | 23 | no |  |  |  |  |  |  |
| Summary pool #3 healthy controls: 5 subjects with median age 45years, 60% males | | | | | | | | | | | |

| **Table S3:**  **Characteristics of subjects from which we generated colon-derived organoid culture.** | | | | |
| --- | --- | --- | --- | --- |
| **IDs** | **Dx** | **age** | **Sex** | **Smoking** |
| 340 | control | 14 | F | no |
| 366 | control | 17 | M | no |
| 367 | control | 6 | M | no |
| 458 | control | 16 | M | no |
| 551 | control | 16 | M | no |

**Methods (extended description)**

**Patient sampling.** Fecal samples, demographics, clinical and laboratory results, and disease characteristics were collected from CD and UC patients and healthy volunteers (10 subjects per group were included in p#1, and 5 per group were included in p#2 and p#3). Fecal samples from UC, CD patients, and healthy volunteers (controls) were selected based on their health index (HI), previously generated by our lab^2^ (i.e., health volunteers with higher indexes), after matching gender and age. Oral samples were collected from 10 healthy subjects. Pools of groups of patients (controls, UC, and CD) were prepared. The pools were aliquoted and stored at -80^o^C for further use (see the scheme in **Fig. 1A)**. Biopsy samples for organoid cultures (n=5) were obtained during endoscopy as part of the routine medical care as previously described^3, 4^. Control biopsies from non-IBD controls were obtained from subjects who were investigated for various gastrointestinal symptoms including abdominal pain but had normal endoscopic and histologic findings. Sheba Institutional Review Board approved the protocol and safety monitoring plan. Informed consent was obtained for each participant.

**Fecal samples preparation.** One gram of each fecal sample was suspended in 10 ml PBS. The mix was then vortexed and put on ice for 5 min. The supernatants were first filtered using 70μm mesh and then 40μm mesh to remove the debris. Samples were centrifuged for 10 min at 3000g and the fecal supernatants (Sup.) containing non-cellular molecules and metabolites were separated from the bacterial pellets. Fecal supernatants (Sup.) were filtered using 0.2um filters. The bacterial pellets were washed 3 times in 1ml PBS and 5 minutes at 2500g. For heat inactivation (heat-killed bacteria, HK), the pellets were then incubated in 1ml PBS at 85^o^C for 60 min for heat inactivation (heat-killed bacteria, HK), followed by centrifugation for 5 min at 1000g. We conducted PCR using 16S primers and were relieved to find no band recovery in the supernatant. In contrast, the PCR results from the HK fraction and the original bacterial fraction showed bands, indicating that there was no 16S DNA present in the supernatant samples. However, it is still possible that small components, like outer membrane vesicles, remained in the supernatant. The pellet debris was discarded and the originating HK fraction was rejoined with the original fecal Sup from the same subject (keeping the same 1:10 ratio between the HK fraction and the fecal Sup). Oral (saliva) samples were processed similarly to feces, starting with ~1 ml of saliva/oral fluid, without adding PBS. The fecal content (joined HK and Sup, 1:10 in PBS) from different individuals was pooled and mixed 1:2 in the media when incubated with Caco-2, and 1:4 in the media when incubated with the colonoids. The oral pool was diluted 1:2 in all experimental assays. For some of the experiments, we used the HK bacteria separately from the fecal Sup, in these cases, we diluted the HK fraction with 1:10 media to keep the same amount as when this fraction is used with the fecal Sup. This was the case in the qPCR, and when we compared the effect between the HK and Sup. fractions. Quantitative PCR (qPCR) was used to quantify 16S V4 DNA bacterial load of samples and pools alongside a quantitative standard curve utilizing standard 305 (ZymoBiomics) using a Fast SYBR Green Master mix (Applied Biosystems, 4385612). DNA was extracted from 20ul processed fecal or oral content samples in 500ul Extract-N-Amp Plant PCR kit (Sigma-Aldrich) extraction/dilution buffer per kit protocol with indicated dilution for measuring the bacterial load (1:16 for fecal and no dilution for oral samples). Bradford protein assay was performed after lysing 200ul of the original, heat-killed, and processed fecal content samples using 100ul RIPA buffer (R0278, Sigma-Aldrich). The resulting supernatant after centrifugation at 14,000 rpm at 4^o^C for 15 mins was collected for the Bradford assay using Thermo Scientific™ Varioskan™ LUX multimode microplate reader with a standard curve running in parallel for BSA. The values obtained for the original and processed samples ranged from 0.8-1.7μg/μl.

**16S rRNA sequencing and analyses**. DNA extraction for microbial characterization was conducted using the direct PCR protocol [Extract-N-Amp Plant PCR kit (Sigma-Aldrich)] ^5-7^. PCRs of the variable region 4 (V4, using 515F/806R primers) of the 16S rRNA were conducted and amplicons were pooled in equimolar concentrations into a composite sample that was size selected (300–500 bp) using agarose gel to reduce non-specific-products from host DNA. Sequencing was performed on the Illumina MiSeq platform at Sheba. Reads were processed in a data curation pipeline implemented in QIIME 2 version 2023.7^8, 9^. Reads were demultiplexed according to sample-specific barcodes. Quality control was performed by truncating reads after three consecutive Phred scores lower than 20. Reads with ambiguous base calls or shorter than 150bp after quality truncation were discarded. Amplicon sequence variants (ASVs) detection was performed using Deblur^10^, resulting in 150 samples with a median of 13,853 reads/sample (IQR, 6526-37021). ASV taxonomic classification was assigned using the 2022.10 Greengenes2 database^11^. For ASVs that did not have an exact match in the database, a naive Bayes-fitted classifier trained on the same 2022.10 Greengenes2 database was used instead. Taxonomy assigned by 16S is indicated by the specific ASV number, and the sequence associated with each ASV number is indicated in **Dataset S1**. All samples were rarefied to 3000 reads for alpha and beta diversity to avoid read number bias. Faith’s phylogenetic diversity^12^ was used as a measure of within-sample α diversity, and unweighted unifrac diversity was used as a measure of sample β-diversity. The resulting distance matrix was used to perform a Principal Coordinates Analysis (PCoA).

Health index and samples prioritization: Samples used included IBD and non-IBD controls, collected at the Sheba Medical Center, and analyzed for microbial composition. A per-sample bacterial health index was calculated as described^2^. Briefly, for each sample, the log of the ratio of health-associated ASV bacteria (97) to disease-associated ASV bacteria (31), was calculated and defined as the health index (with higher values indicating a better health-associated microbiome). The complete list of ASVs used for the health index calculation is listed in Dataset S1. As health and IBD are continuous rather than binary states and in order to get a strong signal for the health-associated bacteria and metabolites, we aimed to prioritize samples from healthy subjects with high health indices. Health index values for all participants are listed in **Dataset S1** (Sheet 1, column B) and are also indicated in **Fig. 1.** Overall, the median (mean) levels of health indexes of subjects were 7.2 (7.3) in the control groups, 0.9 (0.7) in the CD groups, and 4.8 (3.7) in the UC groups.

Multivariate Association with Linear Models (MaAsLin2 R package 1.8.0^13^) was used to identify differentially abundant ASV between IBD and controls, controlling for age and gender as the random variable (ASVs sequences in **Table S1**).

We used the specific ASVs sequences to look for specific potential pathogens:

*Clostridium difficile*: 'TACGTAGGGGGCTAGCGTTATCCGGATTTACTGGGCGTAAAGGGTGCGTAGGCGGTCTTTCAAGTCAGGAGTGAAAGGCTACGGCTCAACCGTAGTAAGCTCTTGAAACTGGGAGACTTGAGTGCAGGAGAGGAGAGTGGAATTCCTAGT

*Salmonella:* 'TACGGAGGGTGCAAGCGTTAATCGGAATTACTGGGCGTAAAGCGCACGCAGGCGGTCTGTCAAGTCGGATGTGAAATCCCCGGGCTCAACCTGGGAACTGCATTCGAAACTGGCAGGCTTGAGTCTTGTAGAGGGGGGTAGAATTCCAGG’
*Campylobacter jejuni*:

TACGGAGGGTGCAAGCGTTACTCGGAATCACTGGGCGTAAAGGGCGCGTAGGCGGATTATCAAGTCTCTTGTGAAATCTAATGGCTTAACCATTAAACTGCTTGGGAAACTGATAGTCTAGAGTGAGGGAGAGGCAGATGGAATTGGTGG

**Metabolomics.** Extraction solution (75% MeOH and 25% ACN and six internal standards) was mixed with the processed fecal samples, vortexed for 10 min, centrifuged at 14,000g for 10 min at 4 °C, and stored at -80c until submission for LC-MS metabolomics analysis. LC-MS analysis was conducted as described^14^. Briefly, Dionex Ultimate ultra-high-performance liquid chromatography (UPLC) system coupled to Orbitrap Q-Exactive Mass Spectrometer (Thermo Fisher Scientific) was used. The resolution was set to 35,000 at a 200 mass/charge ratio (m/z) with electrospray ionization and polarity switching mode to enable both positive and negative ions across a mass range of 67–1000 m/z. The UPLC setup consisted of ZIC-pHILIC column (SeQuant; 150 mm × 2.1 mm, 5 μm; Merck). Stool extracts were injected, and the compounds were separated with mobile phase gradient, starting at 20% aqueous (20 mM ammonium carbonate adjusted to pH 9.2 with 0.1% of 25% ammonium hydroxide) and 80% organic (acetonitrile) and terminated with 20% acetonitrile. Flow rate and column temperature were maintained at 0.2 ml/min and 45 °C, respectively, for a total run time of 27 min. All metabolites were detected using mass accuracy below 5 ppm. Thermo Xcalibur 4.1 was used for data acquisition. Peak areas of metabolites were determined using MZmine2.53^15^ by using the exact mass of the singly charged ions (m/z) and the retention time of metabolites was predetermined on the pHILIC column by analyzing an in-house mass spectrometry metabolite library that was built by running commercially available standards (n=549). Control, UC and CD pools (p#1-3), and Sup. were run in a single batch. Spearman's rank correlation (with dsFDR multiple hypothesis correction threshold of 0.25) was applied using Calour to examine the relationship between individual metabolite abundances and two key metrics: organoid ATP levels and Trans-Epithelial Electrical Resistance (TEER) in the Caco-2 system. Before analysis, metabolite abundance data underwent filtering of spike in metabolites and total sum scale (TSS) normalization, followed by filtering to keep only metabolites significantly different between IBD and control samples (permutation-based non-parametric mean-rank test with dsFDR≤0.1 as implemented in Calour). Metaboanalyst 5.0^16, 17^ was applied for pathway enrichment analyses (Suppl. **Dataset S2**).

For the metabolites positively correlated with ATP and TEER, we aimed to achieve a concentration of the added metabolites in the IBD pools that was slightly higher than what we observed in the control pool. For our initial estimation, we utilized several complementary approaches. First, we conducted a literature review and checked the HMDB database (https://www.hmdb.ca/), but we found only limited data for some metabolites. Next, we estimated the concentration in the pool based on the measured concentration of histidine in fecal samples from healthy volunteers, which is approximately 10uM. We extrapolated other concentrations based on mass spectrometry (MS) peak values. For instance, the azelate peak in the healthy pool was 210, while histidine had a peak of 94. From this, we estimated that the concentration of azelate in feces would also be around 10uM. To validate our estimations, we conducted metabolomics analysis of the fecal pool both with and without the addition of the metabolites at two different concentrations. The actual measurements of the normalized peaks are shown in **Fig. 5D**. The low G-MBs mixture is a close estimate or slightly higher than levels seen in controls, and another mixture with higher metabolite levels was generated to potentially obtain a higher biological effect (“high” G-MBs mixture).

The concentrations used for the five metabolite concentrations were for G-MBs low: Azelate 1uM, pyridoxal 0.1uM, Fructose-6-phosphate 1uM, Galactose 1-phosphate 0.1uM, and Ribose 5-phosphate 0.002uM, and for G-MBs high: Azelate 10uM, pyridoxal 10uM, Fructose-6-phosphate 10uM, Galactose 1-phosphate 1uM, and Ribose 5-phosphate 1uM.

For the metabolites negatively correlated with ATP and TEER, we aimed to achieve a concentration of the added metabolites in the control pools that was slightly higher than what we observed in the IBD pool. The concentrations used for the six metabolites included in B-MBs: Cholate 100uM, Palmitoyl-carnitine 0.02uM, cis-7,10,13,16-Docosatetraenoic acid 1uM, Arachidonic acid 1uM, Histidine 10uM, Taurine 100uM, all-cis-4,7,10,13,16-Docosapentaenoic acid 0.01uM. To validate our estimations, we conducted metabolomics analysis of the fecal pool both with and without the addition of the metabolites. The actual measurements of the normalized peaks are shown in **Fig. S5F**.

**Organoids.** Two biopsies were collected from the recto-sigmoid region during routine colonoscopy and transferred into DMEM-F12+10%FBS with Pen/Strep. The subject’s demographic and clinical information are available in **Table S3**. Crypt-derived organoid preparation was performed as previously described^18, 19^. Biopsies were washed 3 times with DMEM-F12 and Pen/Strep antibiotics. Then, the biopsies were cut into small pieces and incubated with collagenase type 1 (Invitrogen #17100-017) for 15 min` at 37°C, every 5 min` the samples were vigorously processed. The crypts were then seeded with Matrigel (Corning 354234) and L-WRN medium to generate the colonoids. Cells were maintained at 37°C in a humidified atmosphere containing 5% CO2 and were passaged weekly. To maintain proliferation, TGFBIR inhibitor (Biotest- SB431542) is added. To initiate differentiation, EP4 inhibitor (Biotest- L-161,982) is added to DMEM-F12 (Gibco 12634010) medium without FBS. Generating colonoids medium (L-WRN media) was performed as shown previously [Van Dussen- add], using the ATCC mouse fibroblasts cells (L-WRN cells - CRL-3276™), that produce Wnt-3A, R-spondin 3, and Noggin. Organoids monolayer culture was generated as shown previously^20^, with some adjustments. Wells were coated with 100 µL of 2.5% Matrigel (Corning 354234) in cold PBS and incubated for 30min` at 37 °C and 5% CO2. Organoids were expanded for one passage and subsequently collected on day 7. Cell suspensions were obtained by treatment with Trypsin for 2 min` at 37◦C, washed with DMEM-F12, and suspended in the proliferation medium containing 10µM Y-27632 for overnight, until the cells are 100% confluent. Media is then replaced with a differentiation medium for 3 days. Fecal pools (1:4) or inflammatory triggers were added for 5 h. For 3D apical-out colonoid preparation^21^ and treatment. Colonoids were grown for 7 days post-passaging, harvested, and washed with PBS-EDTA (0.5 mM). For apical-out inversion, colonoids were incubated in PBS-EDTA for 30 minutes on ice with gentle rocking, centrifuged (300 × g, 5 min), and resuspended in 50% L-WRN conditioned media supplemented with TGFβRI inhibitor (10 μM) and ROCK inhibitor (10 μM). Colonoids were cultured in 24-well suspension plates for 24 hours to promote polarity inversion. Differentiation was induced by replacing the media with a differentiation-specific medium for 72 hours. On day 3 of differentiation, fecal pools were added to the differentiation medium at a 1:4 (v/v) ratio and incubated for 5 hours prior to sample collection.

**Caco-2.** Caco-2 human colon carcinoma cell lines were purchased from the American Type Culture Collection (Manassas, VA, USA) and maintained in standard culture conditions in DMEM (GIBCO 41965-039, Scotland) containing 20% (for Caco-2) (v/v) heat-inactivated fetal bovine serum (GIBCO 12657-029, Scotland). Cells were maintained at 37°C in a humidified atmosphere containing 5% CO2^22^.

5×10^5^ Caco-2 cells were seeded on 6-transwells plate (Greiner inserts 657630). IFNγ (40ng/ml) and TNFɑ (20ng/ml) were applied at the basolateral compartment, and LPS (100ng/ml) was applied apically. Fecal extract pools were applied apically and incubated for 24 hours (dilution 1:2 in the media). Trans-epithelial electrical resistance (TEER) was determined using Millicell ERS-2 voltohmmeter (Millipore), according to manufacturer’s instruction. Each insert was measured three times (once in each pore) and average values were corrected for background TEER and surface area of the insert to obtain the net-area resistance in Ω∗cm2. TEER measurements were performed 1, 3, 5, 6, and 7 days after culturing. Caco-2 reached full confluency in all experiments. Fecal pools (1:2) or inflammatory triggers were added for 24 h.

**Immunofluorescence Staining.** 3D apical-out colonoids and Caco-2 monolayers were fixed with 4% formaldehyde, washed 3× with PBS, and permeabilized with 0.1% Triton X-100. Samples were blocked in 5% donkey serum and incubated overnight at 4°C with primary antibodies against tight junction protein ZO-1 (anti-ZO1, [Thermofisher 33-9100]), followed by secondary antibodies and/or with Phalloidin-iFluor 488 (Abcam ab176753)). Nuclei were labeled using a DAPI-containing mounting medium. For Imaging, Confocal microscopy was performed using Olympus ix83.

**Cellular ATP level assay.** CellTiter-Glo® Luminescent Cell Viability ATP levels Assay (Promega, G7571) was used as the manufacturer’s protocol. Caco-2 cells and organoids were seeded on a 96-well opaque plate, reached full confluency and intermediate differentiation, and then were incubated for 24 hours with the indicated treatment. The values in each treatment group were then normalized to the untreated control group. For live imaging, control colonoids were incubated with Hoechst (Thermo Fisher 33342, dilution 1:2500) medium on day 3 after starting the differentiation process, and imaged immediately before any additional treatment. Then, Sups of the pools were added, and images were taken at different time points to catch the occurring changes. Imaging was performed using a fluorescent microscope (Olympus ix83) at x4 magnification and a confocal microscope at x20 magnification (Olympus ix83).

**Metabolites cross-validation**. Data from our previously published multi-omics study^1^, which characterized terminal ileum transcriptomics and metabolomics profiles between CD patients and controls, was used to validate the association between 18 metabolites and epithelial health. These metabolites were identified as significantly correlated with epithelial ATP levels or TEER in our culture model. To identify coordinated gene expression patterns, modules of co-expressed genes were constructed using the WGCNA package (version 1.72-1) in R^23^. The correlation between metabolite levels and disease-associated gene modules were then assessed, following established methods^1^. Benjamini-Hochberg FDR correction was utilized only for the metabolites and modules tested here.

***CXCL1.*** For protein secretion, media was collected 24h after the indicated interventional incubation, centrifuged for 5 minutes at 300g, and frozen at -80^o^C till further processing. CXCL1 (R&D Systems, catalog number DY453) levels were measured using specific sandwich ELISA kits according to the manufacturer’s protocol. For cellular *CXCL1* mRNA expression, RT-qPCR was performed using CXCL1-specific primers (forward: 5’-GCAGCAGTCAGTGAGTCTCTTC-3’ and revers: 5’-GGGGACTTCACGTTCACACT-3’). Total RNA was isolated using Tri Reagent-LS (Sigma, T9424, Saint Louis, MO, USA). RNA concentration and purity are assessed on a NanoDrop ND-1000 spectrophotometer (Thermo Fisher Scientific, Wilmington, DE, USA). First-strand cDNA was synthesized using a high-capacity RNA-to-cDNA reverse transcription kit (Applied Biosystems, 4387406). Quantitative real-time polymerase chain reaction (qRT-PCR) is performed using a Fast SYBR Green Master mix (Applied Biosystems, 4385612) and qRT-PCR machine with standard qRT-PCR parameters to analyze the expression of indicated genes compared with the control gene *GAPDH* (forward: 5’-TGGACCTCATGGCCCACA-3’ and revers: 5’-TCAAGGGGTCTACATGGCAA-3’). Results are analyzed with the comparative CT method and relative quantification values [Rq] values. All qRT-PCR reactions are performed in triplicates.

**RT-qPCR.** For mRNA expression *of CXCL8* mRNA expression, RT-qPCR specific primers (forward: 5’-GGAGAAGTTTTTGAAGAGGGCTGAGAAT-3’ and revers: 5’-CAGACCCACACAATACATGAAGTGTTG-3’), DUOX2 RT-qPCR specific primers (forward: 5’- ACGCAGCTCTGTGTCAAAGGT-3’ and revers: 5’- TGATGAACGAGACTCGACAGC-3’), and TGM2 RT-qPCR specific primers (forward: 5’- GGCATGGTCAACTGCAAC-3’ and revers: 5’- CAGCACTGGCCATACTTGAC-3’)

**Statistical analysis.** One-way ANOVA with Šídák's multiple comparisons testing and t-tests were used for normally distributed variables, and the Mann-Whitney U test was used for variables that did not meet the criteria for normality. Categorical variables were reported as frequencies and percentages, with associations analyzed using the Chi-square test. **p*<0.05, ***p*<0.01, ****p* <0.001, **** *p* <0.0001. Analyses were performed with GraphPad Prism v10.1.2.

**Data availability** Processed 16S data are available as **Dataset S1.** The 16S amplicon sequencing dataset was deposited as BioProject PRJNA1216163. Metabolites prioritizations are available as **Dataset S2.**

**References**

1. Braun T, Feng R, Amir A, Levhar N, Shacham H, Mao R, et al. Diet-omics in the Study of Urban and Rural Crohn disease Evolution (SOURCE) cohort. Nat Commun 2024; 15:3764.

2. Abbas-Egbariya H, Haberman Y, Braun T, Hadar R, Denson L, Gal-Mor O, et al. Meta-analysis defines predominant shared microbial responses in various diseases and a specific inflammatory bowel disease signal. Genome Biol 2022; 23:61.

3. Braun T, Sosnovski KE, Amir A, BenShoshan M, VanDussen KL, Karns R, et al. Mucosal transcriptomics highlight lncRNAs implicated in ulcerative colitis, Crohn disease, and celiac disease. JCI Insight 2023.

4. Haberman Y, Tickle TL, Dexheimer PJ, Kim MO, Tang D, Karns R, et al. Pediatric Crohn disease patients exhibit specific ileal transcriptome and microbiome signature. J Clin Invest 2014; 124:3617-33.

5. Braun T, Di Segni A, BenShoshan M, Neuman S, Levhar N, Bubis M, et al. Individualized Dynamics in the Gut Microbiota Precede Crohn's Disease Flares. Am J Gastroenterol 2019.

6. Caporaso JG, Lauber CL, Walters WA, Berg-Lyons D, Huntley J, Fierer N, et al. Ultra-high-throughput microbial community analysis on the Illumina HiSeq and MiSeq platforms. ISME J 2012; 6:1621-4.

7. Braun T, Di Segni A, BenShoshan M, Asaf R, Squires JE, Farage Barhom S, et al. Fecal microbial characterization of hospitalized patients with suspected infectious diarrhea shows significant dysbiosis. Sci Rep 2017; 7:1088.

8. Caporaso JG, Kuczynski J, Stombaugh J, Bittinger K, Bushman FD, Costello EK, et al. QIIME allows analysis of high-throughput community sequencing data. Nat Methods 2010; 7:335-6.

9. Bolyen E, Rideout JR, Dillon MR, Bokulich N, Abnet CC, Al-Ghalith GA, et al. Reproducible, interactive, scalable and extensible microbiome data science using QIIME 2. Nature Biotechnology 2019; 37:852-7.

10. Amir A, McDonald D, Navas-Molina JA, Kopylova E, Morton JT, Zech Xu Z, et al. Deblur Rapidly Resolves Single-Nucleotide Community Sequence Patterns. mSystems 2017; 2.

11. McDonald D, Jiang Y, Balaban M, Cantrell K, Zhu Q, Gonzalez A, et al. Greengenes2 unifies microbial data in a single reference tree. Nat Biotechnol 2024; 42:715-8.

12. Faith DP. Systematics and Conservation: On Predicting the Feature Diversity of Subsets of Taxa. Cladistics 1992; 8:361-73.

13. Mallick H, Rahnavard A, McIver LJ, Ma S, Zhang Y, Nguyen LH, et al. Multivariable association discovery in population-scale meta-omics studies. PLoS Comput Biol 2021; 17:e1009442.

14. Mackay GM, Zheng L, van den Broek NJ, Gottlieb E. Analysis of Cell Metabolism Using LC-MS and Isotope Tracers. Methods Enzymol 2015; 561:171-96.

15. Pluskal T, Castillo S, Villar-Briones A, Oresic M. MZmine 2: modular framework for processing, visualizing, and analyzing mass spectrometry-based molecular profile data. BMC Bioinformatics 2010; 11:395.

16. Pang Z, Chong J, Zhou G, de Lima Morais DA, Chang L, Barrette M, et al. MetaboAnalyst 5.0: narrowing the gap between raw spectra and functional insights. Nucleic Acids Res 2021; 49:W388-W96.

17. Lu Y, Pang Z, Xia J. Comprehensive investigation of pathway enrichment methods for functional interpretation of LC-MS global metabolomics data. Brief Bioinform 2023; 24.

18. VanDussen KL, Marinshaw JM, Shaikh N, Miyoshi H, Moon C, Tarr PI, et al. Development of an enhanced human gastrointestinal epithelial culture system to facilitate patient-based assays. Gut 2015; 64:911-20.

19. VanDussen KL, Sonnek NM, Stappenbeck TS. L-WRN conditioned medium for gastrointestinal epithelial stem cell culture shows replicable batch-to-batch activity levels across multiple research teams. Stem Cell Res 2019; 37:101430.

20. Stroulios G, Stahl M, Elstone F, Chang W, Louis S, Eaves A, et al. Culture Methods to Study Apical-Specific Interactions using Intestinal Organoid Models. J Vis Exp 2021.

21. Co JY, Margalef-Catala M, Monack DM, Amieva MR. Controlling the polarity of human gastrointestinal organoids to investigate epithelial biology and infectious diseases. Nat Protoc 2021; 16:5171-92.

22. Sosnovski KE, Braun T, Amir A, Moshel D, BenShoshan M, VanDussen KL, et al. GATA6-AS1 regulates intestinal epithelial mitochondrial functions, and its reduced expression is linked to intestinal inflammation and less favorable disease course in ulcerative colitis (UC). J Crohns Colitis 2023.

23. Langfelder P, Horvath S. WGCNA: an R package for weighted correlation network analysis. BMC Bioinformatics 2008; 9:559.
